# Similar environments but diverse fates: Responses of budding yeast to nutrient deprivation

**DOI:** 10.15698/mic2016.08.516

**Published:** 2016-08-01

**Authors:** Saul M. Honigberg

**Affiliations:** 1 Division of Cell Biology and Biophysics, University of Missouri-Kansas City, 5007 Rockhill Rd, Kansas City MO 64110, USA.

**Keywords:** pseudohyphal growth, sporulation, meiosis, quiescence, Boolean logic, cell-cell signals, yeast communities

## Abstract

Diploid budding yeast (*Saccharomyces cerevisiae*) can adopt one of several alternative differentiation fates in response to nutrient limitation, and each of these fates provides distinct biological functions. When different strain backgrounds are taken into account, these various fates occur in response to similar environmental cues, are regulated by the same signal transduction pathways, and share many of the same master regulators. I propose that the relationships between fate choice, environmental cues and signaling pathways are not Boolean, but involve graded levels of signals, pathway activation and master-regulator activity. In the absence of large differences between environmental cues, small differences in the concentration of cues may be reinforced by cell-to-cell signals. These signals are particularly essential for fate determination within communities, such as colonies and biofilms, where fate choice varies dramatically from one region of the community to another. The lack of Boolean relationships between cues, signaling pathways, master regulators and cell fates may allow yeast communities to respond appropriately to the wide range of environments they encounter in nature.

## INTRODUCTION

### A) Alternative cell fates: Biology ain’t always Boolean

Most tissues contain multipotent stem cells - i.e. cells able to differentiate into one or more cell types. The choice between fates depends largely on stimuli from the environment/niche of the cell. Often a given fate choice depends on multiple signals - some that promote and some that inhibit a particular fate. These observations suggest that Boolean logic may apply to cell-fate choice - i.e. a particular fate is adopted in response to the presence or absence of a particular combination (or combinations) of signals. For example, the lactose operon is active only when lactose is present and glucose is absent 1. However, many fate choices do not fit easily into the framework of Boolean logic. Given the facile genetics and unmatched gene annotation in *Saccharomyces cerevisiae* 2, this yeast has served for many years as a model for the regulation of differentiation 3,4. The present review focuses on fate choices in diploid cells of the budding yeast, *S. cerevisiae* (Baker’s yeast). I propose that many aspects of this choice are non-Boolean in nature.

**Figure 1:**
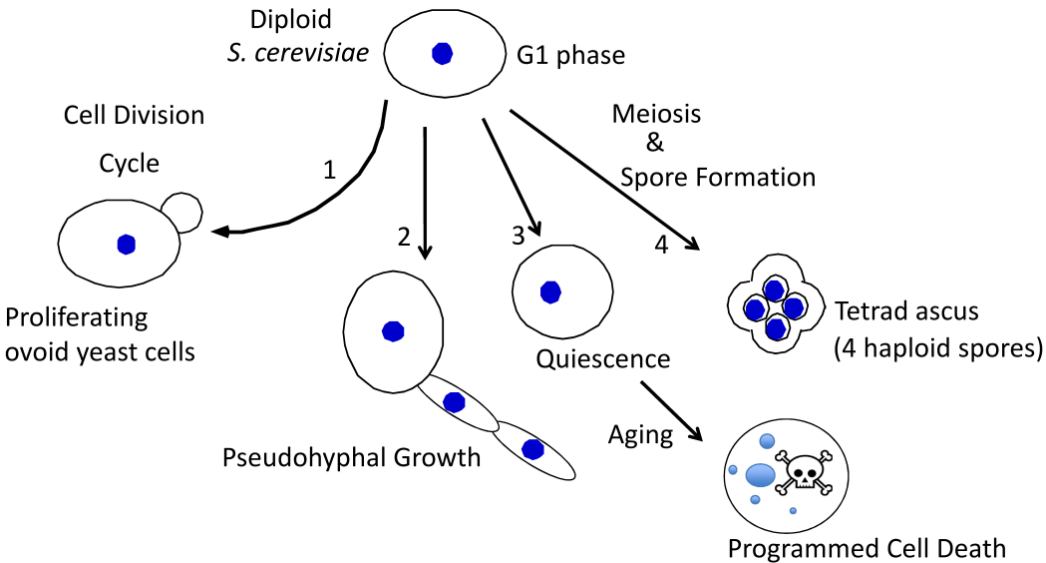
FIGURE 1: Alternative fates for diploid yeast. *S. cerevisiae* typically have an ovoid shape when proliferating (1), and can differentiate to form chains of elongated pseudohyphal cells (2), rounded quiescent cells that subsequently age and succumb to programmed cell death (3), or tetrad asci, i.e. four haploid spores held together in an ascal sac (4).

Diploid yeast can differentiate in multiple ways (Fig. 1). In particular, as nutrients become depleted, these cells differentiate in at least three distinct ways: 1) they can sporulate to form haploid spores (reviewed in 5,6), 2) they can switch into "pseudohyphal growth" (phg) to grow as elongated chains of cells (reviewed in 7,8,9), or 3) they can enter a stable non-proliferative state known as "quiescence" or "stationary phase" where they age and eventually undergo programmed cell death (reviewed in 10,11,12).

The current review focuses on the mechanisms by which *S. cerevisiae* chooses between these several "nutrient-deprivation" fates and the biological functions of each choice. Because in nature individual yeast cells typically proliferate, differentiate, age and die all within the context of multicellular communities such as colonies and biofilms, a particular focus of this review is how cell-fate decisions occur within these communities.

### B) Central hypothesis: Similar environment - different fates

The central hypothesis presented in this review is that the choice of cell fate of *S. cerevisiae* is determined by relatively small differences in nutrient environment, which are then reinforced by cell-cell signals. I term this central hypothesis the "similar environment, different fate (SEDF)" hypothesis.

**Figure 2:**
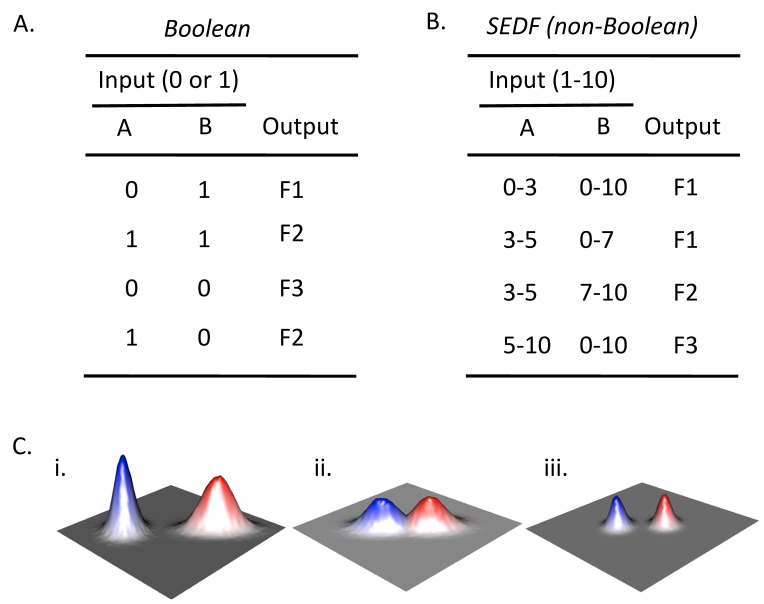
FIGURE 2: Boolean and non-Boolean relationships between input and output. **(A)** Boolean truth table that represents the relationship between all combinations of the presence (1) or absence (0) of two possible inputs (A and B) and the occurrence of a given output. With respect to differentiation choices, examples of inputs could be the presence/absence of particular environmental cues or the activation/ inactivation of particular signaling pathways, and examples of outputs would be the occurrence (1) or not (0) of a particular type of differentiation. In an authentic Boolean truth table the response (as well as signal) would be only "1" or "0", but for the example given, three alternative fates (F1-F3) are indicated for conciseness. As a result, this table can be considered a collapsed stack of truth tables, with one truth table for each possible fate. **(B)** Example of non-Boolean relationship between input and output. Rather than a given input being present or absent, the amount of input affects the output. In the context of differentiation choices, the amount of input could reflect the concentration of a particular environmental cue or the level of activation of a given signaling pathway. Note that in the contrived example shown, when the amount of input A is constant, output depends on the amount of input B not merely its presence or absence (compare row 2 and 3). **(C)** Environmental landscape graphs showing theoretical relationship between the efficiency/probability of cell fate (Z-axis) and two environmental variables (X- and Y-axes). The red and blue peaks represent two different cell fates. (i) In a Boolean landscape, fates are discrete, they never occur in the same environment, also Boolean response peaks are symmetric relative to the axes, so the blue peak is Boolean and the red peak is not. (ii) SEDF model is not Boolean since the two fate response peaks overlap. (iii) Even in the SEDF model, fates can be made discrete by reinforcing small differences in environment by cell-cell signaling.

The SEDF hypothesis contrasts with a view in which each cell fate responds to discrete differences in environmental cues. Cell-fate decisions determined by discrete differences in cues can be expressed a Boolean relationship between these cues and a given cell fate. An example of a Boolean relationship between inputs and outputs is shown in Fig. 2A. Boolean logic requires that there are two states for each input (e.g. "1" and "0") with respect to environmental cues. For example, if a response is linked to a threshold level (e.g. if a given fate requires the presence of a nutrient above a certain concentration), that would also be considered Boolean, since there are effectively only two states. An example of a non-Boolean relationship between input and output is shown in Fig. 2B. In this example the range of concentrations of a given cue that activate a particular cell fate depends on the concentration (not simply the presence or absence) of a second cue. Thus, the key feature of SEDF is that the relative level of multiple cues determines cells fate, not just their presence or absence.

Either a Boolean or non-Boolean model is consistent with the observation that each fate occurs most frequently in some environments than others. However, in a Boolean model, as mentioned above, the environments that promote one fate are clearly discrete from the environments that promote a different fate. A Boolean relationship is represented by a theoretical landscape (Fig. 2Ci). The discrete red and blue peaks in this figure represent two discrete differentiation responses; the two axes represent increasing intensity of two environmental cues (e.g. increasing concentration oxygen and nitrogen). In contrast, in a non-Boolean model the environments that promote each fate can overlap (Fig. 2Ci).

The landscape corresponding to the “red fate” in Fig. 2Ci shows an additional feature not allowed in Boolean models. Unlike the blue peak, in the red peak the two signals interact such that the response peak is not symmetrical relative to the axes. In other words, the optimal level for one cue is different, depending on the level of the second cue.

There are three main reasons to propose yeast cell fate follows an SEDF (non-Boolean) rather than a Boolean model, as discussed throughout this review. First, all 3 types of diploid differentiation occur in very similar environments, so fate choice is probably determined not by the presence or absence of one or more extracellular signals, but by the relative amount of these signals, i.e. fate choice cannot be represented by Boolean logic. Second, the transcription factors and signal transduction pathways that regulate yeast cell differentiation are not, either alone or in combination, specific for only one form of differentiation. Indeed, not only is the relationship between fate choice and environmental cues not Boolean, neither is the relationship between fate choice and the activity of most regulators. Third, yeast differentiation fates, despite mechanisms ensuring their stability, are remarkably flexible without dramatic changes in environment. For example, many communities of yeast are partitioned into populations undergoing different fates.

The main ramification of the SEDF hypothesis is to underline the importance of cell-cell signals in the context of yeast communities. As proposed below, the key mechanism that allows SEDF is cell-cell signaling that reinforces fate choice. Within these communities, the combination of environmental and cell-cell signals allows cell fate choice to be coordinated both temporally and spatially. In particular, I propose that relatively modest quantitative differences in nutrient environment (and signal pathways activity) are sufficient to efficiently specify a single cell fate because these small differences are reinforced by cell-to-cell signals. In the context of the theoretical landscapes described above, one can consider that these cell-cell signals allow discrete fate peaks even in a very similar environment - the effectively "sharpen" these peaks (Fig. 2Ciii). As a result, SEDF allows different regions of the community to adopt complementary fates within a relatively uniform environment. Furthermore, this type of coordination provides biological functions to the community that are not available to individual cells. As a result, these signals provide functions that may echo the origins of communication on this planet.

## LESSONS FROM THE WILD 

Before considering the evidence for SEDF, it is useful to regard possible differences between yeast found in nature (in which fate choices evolved) and yeast in the laboratory (in which fate choice can be studied). Recently, it has become clear that natural populations of *S. cerevisiae* exist throughout the world 13,14,15 and that these natural populations are different from domesticated yeast populations such as industrial or vineyard yeast as well as from clinical yeast isolates 16,17. In particular, the ability to isolate, genotype, and in many cases sequence the genome of these natural isolates of *S. cerevisiae* has revealed at least two important features of the evolution of cell fate choice in wild yeast - the stability of the diploid state and the diversity of ecological niches in which these yeast can be found.

### A) Natural *S. cerevisiae* populations are homozygous diploids

Based on genome analysis of many wild yeast isolates, it is clear that the ploidy of *S. cerevisiae* isolated from natural environments is diploid rather than haploid or polyploid 18,19,20. By way of contrast, in the lab *S. cerevisiae* readily proliferates as haploids, diploids or polyploids. Indeed, polyploidy (particularly tetraploidy) is very common among industrial and food-processing *S. cerevisiae* 21,22, and polyploidy and even aneuploidy may occur relatively frequently in response to environmental stress (reviewed in 23).

The fact that haploid yeast have not been isolated from the wild does not necessarily mean that sporulation is rare in natural environments. Indeed, yeast strains isolated from nature generally sporulate and mate efficiently in the laboratory under a range of nutrient conditions 24,25,26. However, any spores isolated from nature might not be recognized as such, since they would probably germinate, mate and form diploids as soon as first cultured in the lab.

There are several reasons that haploids are likely short-lived intermediates both in nature and when first cultured in the lab. In the first place, after sporulation is complete, the four haploid products of sporulation, two of each mating type, are held tightly together in an ascal sac - the remnant of the cell wall of the parent cell. As a result, once nutrients are restored, haploids of opposite mating type efficiently undergo mating with other spores from the same ascus (intra-ascal mating) to restore the diploid state. Furthermore, any haploids that fail to mate with their sister spores would likely mate soon after beginning to proliferate, because most wild yeast are homothallic, meaning that a dividing cell produces a daughter cell of the opposite mating-type from the mother cell, allowing subsequent mating between mother and daughter. Finally, diploid cells proliferate more rapidly than haploid cells under most conditions 27, allowing diploids to out-compete haploids in the wild. The greater stability of the diploid state in *S. cerevisiae* contrasts with other yeast species such as *S. pombe* and *Candida lusitaniae*, which are both more stable in the haploid state. In these species, meiosis closely follows mating, rather than the reverse as in *S. cerevisiae* 28,29.

One implication of intra-ascal mating in wild yeast is that inbreeding between sister spores is much more frequent than outbreeding of unrelated yeast 30. Also contributing to the low rates of outcrossing is the fact that yeast grows and sporulates primarily in clonal communities, i.e. starting from a single cell. Consistent with high levels of inbreeding in natural populations, analysis of molecular polymorphisms including whole-genome sequencing of natural isolates reveal them to be largely homozygous. Indeed, natural yeast have significantly less heterozygosity than clinical or vineyard isolates 31,32 perhaps because of less selection pressure from human associations on the natural yeast. Although inbreeding may be the rule for wild yeast, genome-wide sequencing indicates that outbreeding does occur 30 including between wild and domesticated yeast 13. For example, outbreeding may occur in the gut of insect vectors such as wasps 33. In fact, it has been suggested that the ratio of inbreeding to outbreeding can be regulated in wild yeast 34.

Haploids may be rare in the wild, but their ability to proliferate in the laboratory is extremely useful. Haploids can be easier to work with than diploids; for example, only a single allele must be deleted to eliminate the gene product. Furthermore haploids undergo many of the same fate choices as diploid cells. For example, haploids can switch from budding to filamentous growth, and haploids can enter quiescence and age. Although wild-type haploids are not normally able to initiate meiosis & sporulation, introduction of certain mutant alleles can bypass these constraints 35,36.

To return to the evolution of fate choice, since yeast found in the wild are almost always largely homozygous diploids, it is clear that the nexus for yeast differentiation is the diploid cell as nutrients become depleted. Below I describe how both yeast’s metabolism and its natural habitats provide the environmental cues that trigger differentiation.

### B) Natural *Saccharomyces* habitats are both diverse and changeable

It is useful to consider the natural habitats/ecological niches of yeast in the wild with respect to the biology of fate choice. In the lab, yeast differentiate when nutrients becoming limited, not only because nutrient depletion slows or halts proliferation, but also because it directly promotes differentiation. As discussed in this section, it is particularly the changes in metabolism and hence nutrient environment accompanying late stages of growth that promote differentiation.

The crux of *S. cerevisiae* metabolism is the "Crabtree effect" 37. The Crabtree effect describes the observation that when glucose is available, yeast will metabolize (ferment) this sugar completely to ethanol, acetate and other non-fermentable carbon sources (NFCs), and these non-fermentable products are only themselves efficiently metabolized further (via respiration) once glucose is completely exhausted. This switch from fermentation to respiration, termed the diauxic shift, occurs even when oxygen remains plentiful throughout. Not surprisingly, while glucose is plentiful, multiple signal transduction pathways respond to this glucose to repress respiratory enzymes and metabolism of alternative carbon sources (reviewed in 38). Thus, *S. cerevisiae* is primarily adapted to proliferate on glucose and convert it to non-fermentable carbon.

Many yeast species do not display the Crabtree effect, and it has been suggested that this effect evolved in *S. cerevisiae* and other "Crabtree yeast" as a mechanism to out-compete surrounding microbial species because the ethanol produced by fermentation and secreted by the yeast inhibits the growth of these other species 39. The Crabtree effect is also central to the economic power of *S. cerevisiae* because it allows the production of high levels of ethanol during fermentation and high levels of CO_2_ during respiration.

Wild yeast have been observed to proliferate in nature primarily on plant matter rich in sugars, such as tree exudates or rotting fruit 14,26. In contrast, recent isolation of *S. cerevisiae* from sites throughout the world such as soil demonstrates that this species is capable of inhabiting a wide range of ecological niches that are not rich in glucose 15,40. It seems likely that in these latter niches, yeast is not proliferating and may exist primarily as spores.

**Figure 3:**
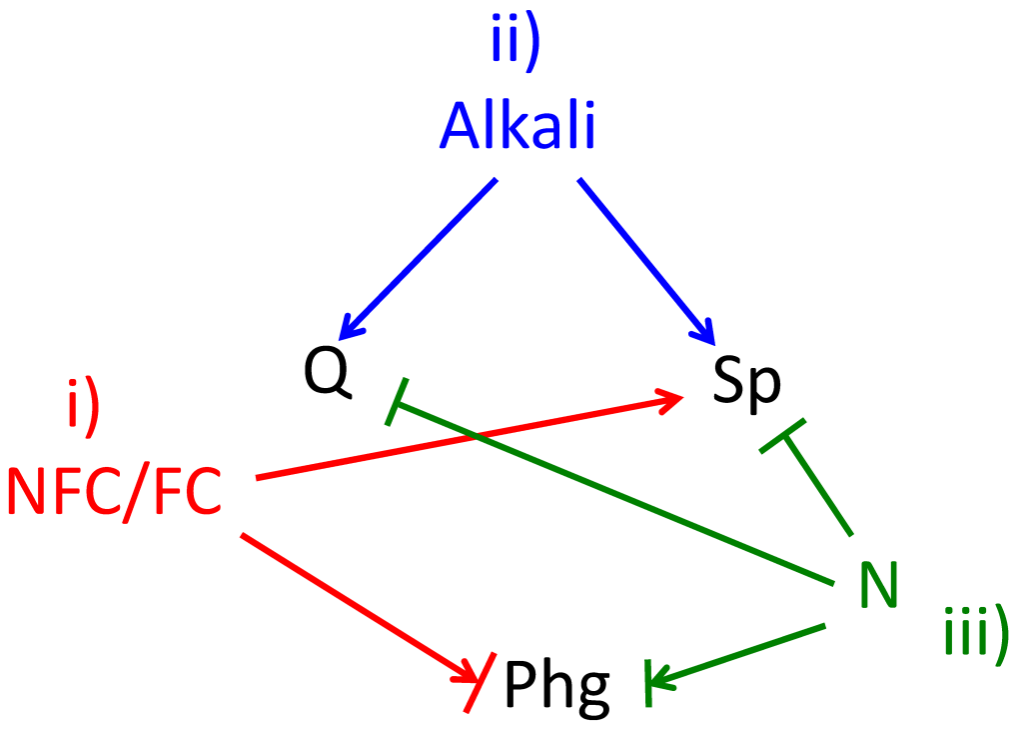
FIGURE 3: Environmental cues determine cell fate. All three differentiation fates occur as nutrients become depleted, and this depletion provides at least three environmental cues that control differentiation fate as follows.**(i)** The ratio of non-fermentable carbon sources to fermentable carbon sources (NFCs/FCs) affects fate, with higher levels of NFCs stimulating sporulation (Sp). The arrow + bar shown linking NFCs/FCs to pseudohyphal growth (phg) reflects that in some laboratory strain backgrounds phg occurs efficiently when a FC source (glucose), is present, whereas in other strain backgrounds this differentiation occurs more efficiently in a NFC (acetate).**(ii)** Alkali increases in the environment during late stages of growth, and this alkali promotes both quiescence (Q) and sporulation. **(iii)** Nitrogen and possibly other essential nutrients (N) inhibit both, sporulation and quiescence. The arrow bar connecting N to phg represents that phg occurs most efficiently when intermediate levels of N are present, i.e. Phg is inefficient at either high N or in the absence of N.

Nutrient environment changes rapidly both during late stages of growth and during differentiation itself. During late stages of growth, fermentable carbon sources, nitrogen and other nutrients become depleted whereas NFCs can remain relatively plentiful (Fig. 3). Indeed, both the intracellular and extracellular metabolome studies reveal dramatic changes in the concentrations of intermediary metabolites and amino acids during the diauxic shift 41,42. These changes continue as differentiation progresses. For example, analysis of sporulation in transcriptome 38,43, proteome 44,45, and metabolome 46,47 studies indicate that the expression and activity of metabolic enzymes continues to fluctuate as sporulation progresses.

In summary, the biology and natural ecology of yeast indicate that yeast is distributed widely, but that it may primarily proliferate on fermentable carbon sources (FCs). The Crabtree effect ensures that as cells begin to exhaust nutrients and slow or cease growth, NFCs remain relatively plentiful. As discussed in the Section "Shared environmental cues & distinct fates", this nutrient environment is optimal for differentiation regardless of the particular fate (consistent with SEDF). Thus, both the metabolism and the ecology of yeast suggest that yeast evolved such that late stages of growth provide the environmental cues necessary for differentiation.

### C) A caveat regarding comparing laboratory and natural strains 

Regulation of cell differentiation by nutrient environment has been studied in many different laboratory strains of yeast. It is now evident that these strain backgrounds can vary significantly with respect to the relationship between environmental cues and differentiation fates. These results raise the question of how well fate choice in lab strains reflects the fate choices that evolved in the wild. Indeed, strain variants with altered differentiation responses may have been selected in early laboratory strains, descendants of which are now used in most modern laboratories 48. The effect of strain backgrounds on differentiation responses is a critical consideration when synthesizing data from different studies done in different strain backgrounds.

One example of phenotypic variation between common laboratory strain backgrounds is sporulation efficiency. Moreover, this variation extends to different isolates of industrial, clinical and wild yeast. Among some of these strains, variation in sporulation efficiency has been traced (e.g. by QTL analysis) to allele differences at a relatively few loci, e.g. the transcription factor Rme1 32,49,50. Furthermore, sporulation efficiency under a single condition likely underestimates the variation in sporulation capacity between strains. As one example, several common lab strain backgrounds (e.g. W303 and SK1) sporulate very efficiently under optimal conditions but sporulate much less efficiently than natural isolates on low concentrations of glucose 25.

Phg efficiency also varies considerably between strain backgrounds - both among laboratory strain backgrounds and among natural isolates of yeast 51. In the strains that have been compared, this variation is again largely attributable to one or a few loci; for example, the transcription factor Flo8, a master regulator of phg 52,53,54. In fact, several common laboratory strain backgrounds of yeast that lack a functional allele of Flo8 are completely unable to undergo phg, but can partially recover this ability when Flo8 is restored 55. As with sporulation, variation in phg extends beyond efficiency under optimal conditions. For example, in some laboratory strain backgrounds (e.g. Ʃ1278b) phg occurs most efficiently in medium containing glucose, whereas in other strain backgrounds (e.g. SK1), phg occurs most efficiently in NFCs 56,57,58,59. Finally, as with phg and sporulation, the rate of ageing in quiescent cells varies significantly between different laboratory strains 60.

In addition to allele variation for master regulatory genes such as *RME1* and *FLO8*, the responsiveness of signal transduction pathways to environmental cues varies significantly between laboratory strains (reviewed in 61). Many experiments connecting signaling and differentiation pathways have been done in only a single strain background, so it is wise to be circumspect in synthesizing results based in different strain backgrounds. An equally important point is that it is unlikely that any single laboratory strain background represents the "real" evolved response; indeed, the variation between laboratory strains is mirrored by variation between natural isolates.

Obviously, experiments with lab yeast strains have driven and will continue to drive most of what we understand about fate choice, as they do for most yeast biology. However, in considering the implications of these experiments to fate choice, it is useful to remember possible differences between these lab strains and the natural yeast strains in which fate choice evolved.

## VARYING FATES, VARYING FUNCTIONS

From a functional viewpoint, a key aspect of the choice between cell fates is that each fate has a different biological role. In this section, these roles are discussed, especially in the context of the nutrient limitation that triggers diploid yeast differentiation.

### A) Fate #1: sporulation - sex, food and energy

The primary function of sporulation is to produce cells (haploid spores) that are more resistant to environmental stresses than the vegetative cells from which they derive. For example, spores resist antimicrobials, high temperatures and prolonged starvation to a much greater extent than vegetative cells 62,63,64. This resistance derives in large part from the thick walls encasing each spore 65,66. Another aspect of spore resistance is that spores survive the insect gut much better than vegetative or quiescent cells 62. Fruit flies and other insects are thought to be a major vector by which yeast spread from one ecological niche to another and outbreed to less related strains 67,68. Indeed, it has been proposed that the environment of the insect gut is the primary site at which the spore wall protects viability 5.

Another presumed function of sporulation is to increase genetic diversity in a yeast population as a result of meiotic recombination coupled with independent assortment of chromosomes. Indeed, meiotic recombination in yeast may also increase genetic variation as a result of increased mutation rates near meiotic recombination sites 69. However, as mentioned earlier, most wild yeast communities are clonal populations of largely homozygous strains. Yeast meiosis could be important in generating diversity after relatively rare out-breeding or mutation events by stimulating loss-of-heterozygosity at new alleles. In any case, yeast meiosis likely provides a relatively minor selective advantage relative to the increased environmental resistance of spores.

Sporulation as a response to limiting nutrients is particularly interesting in the context of the large energy requirement for the sporulation program. For example, energy is required to express hundreds of gene products required for meiosis and spore wall formation including many, such as spore wall proteins, that are produced to very high levels 44,45,70,71,72. In addition, many of the cellular processes required in sporulation have additional energy requirements - e.g. DNA replication and chromosome segregation. This abundant expenditure of energy in response to nutrient limitation has been termed the sporulation "energy paradox" 73,74.

It is likely that this apparent paradox is resolved through a combination of several mechanisms. In the first place, storage carbohydrates, for example glycogen, accumulate during late stages of growth and are subsequently utilized for energy during sporulation (reviewed in 75). Indeed, mutants defective in accumulating these storage carbohydrates fail to sporulate 76,77. In the second place, deprivation for nitrogen (or other essential nutrients) when NFC is still abundant allows abundant energy production/respiration in the absence of cell division. Thus, yeast build environment-resistant spore walls by using both internal and external energy sources generated during growth.

### B) Fate #2: Quiescence - dormancy, ageing & death 

#### 1) Not dead, just quiet.

 Quiescence is a differentiated state in which yeast cease growth (reviewed in 12) and undergo genome-wide changes in transcriptional expression and chromosome topography 78,79,80, cytoskeletal organization 81 and cytosolic fluidity 82,83. Thus, like sporulation, quiescence is a response to nutrient deprivation that is likely to require an energy investment. One of the major functions of quiescence is the same as that of sporulation - resistance to environmental stresses. For example, activation of the Mpk1 cell wall integrity pathway in quiescent (Q) cells induces the induction of cell-wall repair genes 84,85, and activation of Rim15 kinase in these same cells induces stress-resistance genes 86,87,88. Quiescence in yeast has been studied primarily in haploids but is equally available to diploid cells.

Interestingly, Q diploids are more resistant to environmental stress than growing cells but less resistant than spores to environmental stress 5. Q cells do not remain viable indefinitely. As time passes and Q cells age, their viability diminishes. Thus, quiescence, aging, and eventual death can be considered progressive stages in a single differentiation pathway.

#### 2) The universal fate choice - getting older vs. the alternative.

 As stated above, cell death occurs naturally as cells age. For example, in suspended yeast cultures most cells have reached the end of their lifespan approximately 1-3 weeks after they have ceased growth 63,89,90. Lifespan is ended through a programmed cell death (PCD) (reviewed in 91,92,93).

PCD in yeast displays many of the same cellular landmarks as apoptosis in higher organisms, such as DNA fragmentation, cell surface changes, and involvement of mitochondria and reactive oxygen species (ROS) 94,95. For this reason, yeast PCD is often referred to as "yeast apoptosis". However, yeast PCD does not utilize all of the same regulators as mammalian apoptosis nor the extensive family of caspases typical of metazoan apoptosis 96,97. In this review, to avoid semantic distinctions, I refer to yeast apoptosis as PCD.

Yeast lifespan is limited not only by the period of time that elapses after growth ceases (chronological ageing), but also by the number of times a mother cell can divide before it dies (replicative ageing) (reviewed in 98). These two types of ageing are regulated by many of the same pathways (reviewed in 99,100), and they are also linked in the sense that chronologically aged yeast have shorter replicative lifespans than chronologically young cells 101. Nevertheless, the two types of age are not interchangeable; for example, the Sir2 histone deacetylase inhibits replicative ageing, but Sir2 actually stimulates chronological ageing in some strain backgrounds and conditions (reviewed in 102).

In addition to ageing, yeast PCD is also triggered by a wide variety of other environmental stresses 103,104,105,106. A common feature between ageing and most other triggers for PCD in yeast is the accumulation of oxidative and other cellular damage. According to one view of ageing, accumulation of damage over time eventually triggers PCD (reviewed in 107).

What is the relationship of nutrient environment to ageing and subsequent cell death? A common feature of replicative lifespan control from yeast to metazoans is that lifespan is increased when nutrients are limited, i.e. calorie restriction 108,109. Although the role of calorie restriction in lifespan extension is still a matter of debate, one idea is that when metabolism is limited, ROS and hence oxidative damage are also limited, and as a result lifespan is extended 110,111,112,113. A corollary to this hypothesis is that inducing repair of oxidative stress also extends lifespan 114,115.

Regardless of the of PCD trigger, its function in yeast and other single-celled organisms has been the subject of debate. One idea is that because yeast growth is largely clonal, programmed death in one cell could benefit other cells with the same genotype, thus providing a selective advantage for the genotype. For example, suspended cultures accumulate ROS after prolonged incubation, triggering PCD in most of the culture and presumably releasing enough nutrients from the dying cells to allow a subpopulation of still viable cells to continue growth and acquire adaptive mutations 116. More generally, several investigators have proposed mechanisms by which programmed cell death benefits the overall (or average) survival of a clonal community in *S. cerevisiae* 92,117 and other microorganisms 118,119,120. The specific case of cell fate choice (including PCD) within communities is discussed further in the section "Shared communities – coordinated fates".

In summary, quiescence, ageing and PCD can be considered a single progressive pathway with different functions at earlier stages (e.g. resistance to environmental stress) than at later stages (e.g. possible re-distribution of nutrients).

### C) Fate # 3: Phg- the life of the forager

#### 1) Overview: looking for a better neighborhood.

Phg, like both quiescence and sporulation, is a response to diminished nutrients. However, phg is unique among yeast diploid differentiation fates in that it is also a means of cell proliferation.

One likely function of phg is foraging. Specifically, phg allows yeast communities such as colonies to expand and access distant nutrients more efficiently than is possible when yeast divide through its standard (ovoid) budding patterning. Indeed, in many strain backgrounds phg occurs only in communities, not in suspended cultures 121. However, in other strain backgrounds pseudohyphae will form under certain conditions even in suspended cultures 122,123.

At least two types of foraging are associated with pseudohyphae: i) extension of chains of elongated yeast cells along the surface of the underlying substrate, and ii) invasion of these chains into the underlying substrate.

#### 2) Exploring the surface.

The first type of foraging is closely related to the structure of pseudohyphae as chains of elongated cells. These chains radiate out from the perimeter of a colony or biofilm along the surface of the agar or other hard surface like plastic on which these communities grow. Limiting phg to the fringe of the community may be the most efficient mechanism to access distant sources of food.

#### 3) Exploring below the surface.

As yeast colonies mature, they sometimes grow into (or "invade") the underlying agar medium. Although invasive growth is often studied in haploids, diploids colonies are equally capable of invading agar. By the same reasoning as above, invasive growth potentially allows access to distal nutrients. In addition, invasive growth may provide benefits by anchoring a yeast community to its underlying substrate, and invasive growth of yeast into fruit and other natural substrates have been observed 124. By analogy, the much longer hyphal and pseudohyphal filaments formed by the pathogenic yeast *Candida albicans* are necessary for these yeast to invade host tissue and, hence, for pathogenicity (reviewed in 125).

In theory, the elongated-chain geometry of pseudohyphae could provide the force necessary for invasion, but in fact the connection between pseudohyphae and invasive growth is not straightforward. Most laboratory strains that can form pseudohyphae can also invade agar 57,59,126. However, many genes have been implicated in one program but not the other 127,128. Furthermore, in some wild and laboratory yeast grown on agar plates, the region where colonies invade the agar is not associated with extensive phg 25.

#### 4) Phg and phg-spectrum phenotypes.

Invasiveness is only one of a spectrum of wild-type phenotypes that require many of the same genes as phg but which do not always directly require phg. In particular, these "phg-spectrum" phenotypes all depend on expression of flocculins, which are a class of lectin-type proteins involved in both cell adhesion and cell signaling 129. The diversity of flocculin-dependent phenotypes reflects the variety of communities that can form in this species. For example, the *FLO11* flocculin is required to form "structured colonies", which have with a distinctive lacey appearance 130,131, "mats", which are large thin colonies formed on low agar (high moisture) plates 132, "flors", which are thin colonies of yeast that form on the top of liquid cultures 133, "flocs", which are large clumps of cells that form suspended in cultures 58,134, and "minicolonies" which are biofilm like structures that adhere to plastic surfaces submerged in medium 56,133. Both lab and natural yeast isolates vary considerably in their ability to undergo these phg-spectrum phenotypes 20,131.

#### D) Summary - distinct functions for discrete fates

As discussed in the Section "Lessons from the wild", all three differentiation fates are a response to a limited-nutrient environment, but as we saw in the Section "Varying fates, varying functions", each fate has distinct functions and costs from the other two cell fates. For example, both quiescence and sporulation result in cells that are more resistant to the environment, but the higher resistance of spores relative to quiescence comes at the expense of a sizeable energy investment. Phg, a response to less severe nutrient depletion than the other two fates, functions more for foraging than for resistance to stress. Because fate choices are functionally quite distinct, it is striking, as discussed below, that they all respond to many of the same environmental cues, signaling pathways, and master regulators.

### OVERLAPPING REGULATORS BUT DISTINCT FATES

#### A) Introduction to the master regulators of differentiation 

**Figure 4:**
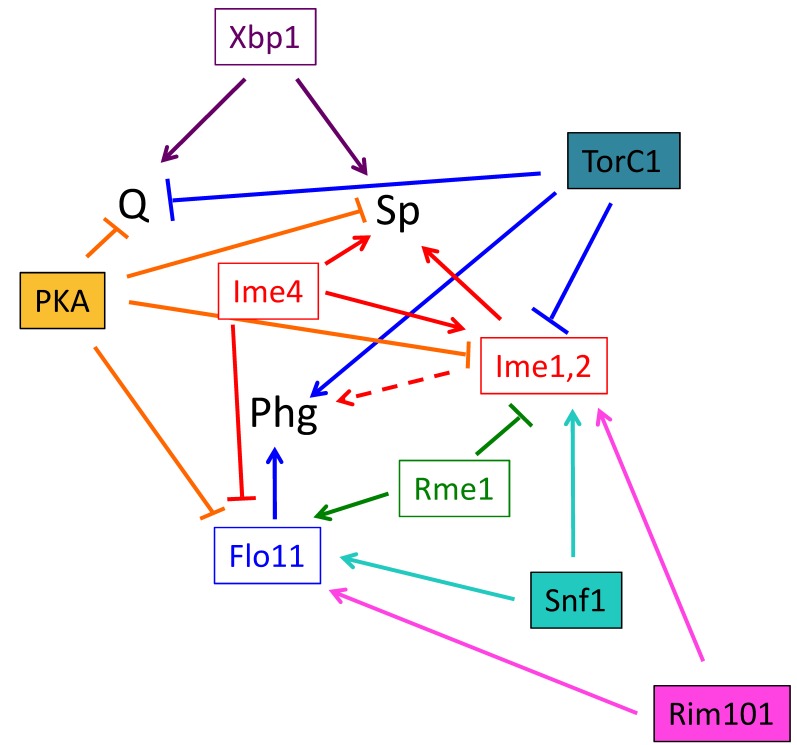
FIGURE 4: Roles of signal transduction pathways and master regulators on cell fate. Known relationships between signal transduction pathways (filled rectangles), master regulators (open rectangles), and cell fate (abbreviations as in Fig. 3). The dotted arrow connecting the Ime1 and Ime2 master regulators (Ime1, 2) and phg represents that these master regulators are required for phg in some strain backgrounds but not in others. It should be noted that this diagram is meant as a working model of these relationships, other connections between pathways and regulators are likely.

At one level, differentiation fate is determined by expression of master regulator(s). Master regulators are gene products that are required to initiate differentiation; indeed by one definition, a true master regulator is sufficient to trigger differentiation when expressed ectopically under conditions where that fate is normally suppressed. In a Boolean logic system, each fate would be defined by the presence or absence of one or more master regulators. Indeed, each differentiation pathway in yeast requires one or more master regulators and subsequent expression changes in hundreds of genes. However, as described below several of these master regulators activate more than one differentiation pathway, repress more than one pathway, or activate one pathway while repressing another (Fig. 4).

Phg is regulated by a set of transcription factors including Flo8, Ste12, Tec1 and Nrg1, all of which activate transcription of the flocculin gene *FLO11* (reviewed in 135). Sporulation is initiated by expression of the Ime4 RNA methylase, the Ime1 transcription factor and the Ime2 protein kinase (reviewed in 5). Quiescence is activated by expression of the Xbp1 transcription factor 78,79.

#### B) Activators of meiosis (*IME1, IME2*) also activate phg 

Surprisingly, two key master regulators of sporulation, the Ime1 transcription factor and Ime2 kinase, are also required for phg in some laboratory strain backgrounds. In particular, these genes are required for phg in the SK1 but not the Ʃ1278b background 57. This result is consistent with an observation described above - in SK1, phg (like *IME1* and *IME2* expression) is induced by NFC, but in Ʃ1278b phg is induced by glucose, which strongly inhibits *IME1* and *IME2*.

The roles of Ime2 in activating both phg and sporulation in SK1 is broadened further when Ime2 homologs in other fungal species are considered. For example, Ime2p homologs in *Aspergillus nidulans* and *Neurospora crassa* activate the development of sexual structures, and Ime2 homologs in *Ustilago maydis* and *Cryptococcus neoformans* activate mating and filamentous growth (reviewed in 136). Thus, the idea that a master regulator can control more than a single differentiation program extends across species.

#### C) Rme1 and Ime4 activity and the sporulation/phg choice 

Rme1 is a transcriptional activator that inhibits sporulation and stimulates phg. Rme1 prevents sporulation in haploid cells, a critical function since haploid meiosis is inevitably lethal. Rme1, which is expressed at high levels in haploids exposed to sporulation conditions, prevents meiosis by activating transcription of *IRE1* a non-coding gene approximately 1 kb upstream of *IME1*. Transcription of *IRE1* through the *IME1* promoter prevents *IME1* transcription 137.

At the same time that Rme1 blocks *IME1* transcription in haploids, it also activates *FLO11* transcription to promote filamentous growth 138. Rme1 is also expressed in diploids (though at lower levels), and hence may balance sporulation and pseudohyphal fates in diploids. For example, wild yeast isolates with relatively high Rme1 expression tend to have low sporulation and high phg, whereas strains with relatively low Rme1 expression have the reverse tendency 32.

Not surprisingly, both *IME1* and *FLO11* are regulated by a number of other transcription factors in addition to Rme1. Indeed, *IME1* and *FLO11* have among the largest upstream intergenic distance of any yeast gene - consistent with these genes having especially complex promoters 139. Multiple nutrient signals are integrated in regulating *FLO11* not only through the transcription factors that bind its complex promoter but also through regulating activity of the Msb2/MAPK pathway that activates some of these transcription factors 135.

The expression pattern of *IME4* is opposite to that of RME1 *IME4* is expressed to higher levels in diploids than in haploids 140. Like *IME1*, *IME4* is regulated by an overlapping long noncoding transcript 141,142. Furthermore, Ime4 also acts opposite to Rme1 in balancing sporulation with phg. For example, Ime4 inhibits *FLO11* transcript accumulation while promoting Ime1 transcript accumulation 141,143. Ime4 is an RNA N-6 adenosine methyltransferase that likely acts on many hundreds of RNAs 144. Interestingly, N-6 adenosine methylation also regulates expression of large gene sets in plants and animals during differentiation and development (reviewed in 145). Other proteins that (like Ime4) promote sporulation and inhibit phg include Bir1, which is homologous to IAP (inducer of apoptosis protein) 146, and Spo21, a 67 aa protein that localizes to the prospore 147.

#### D) Quiescence & sporulation: shared regulator and shared properties

Xbp1 represses transcription of many genes by targeting the Rpd3 histone deacetylase to these genes 79. Interestingly, Xbp1 is not only required for quiescence but also for sporulation, at least in part because Xbp1 represses transcription of G1 cyclins 148,149. These cyclins not only trigger the G1 to S transition, they also repress *IME1* transcription and inhibit Ime1p nuclear localization 150,151. It is not known whether Xbp1 is required for all forms of quiescence.

Most meiotic genes are not induced in quiescent cells, but at least some of the same metabolic enzymes and stress resistant enzymes are induced in both programs 46,152. For example, trehalose synthesis is required for both initiation of meiosis and maintenance of quiescent cell viability 76,153. Similarly, many proteins required for heat shock resistance are induced during both quiescence/ageing and sporulation 44,154,155. It is possible that cells first enter quiescence and only then may sometimes also initiate sporulation. Alternatively, quiescence and sporulation may be mutually exclusive fates that share certain common regulators and target genes.

#### E) Summary 

Master regulators of diploid cell fate do not display Boolean-logic relationships to fate choice, i.e. there is not a particular combination(s) of known master regulators that specify each fate (Fig. 4). Instead, the relationship between master regulators and cell fate is complex. Indeed, some regulators, such as Flo11 regulate only a single fate, whereas other regulators (Xbp1, Ime1, and Ime2) activate more than one fate. Finally, a third class of regulators (Rme1 and Ime4), activate one fate while repressing another. How these master regulators together determine fate choice is unknown, but evidence so far indicates that their relationship to fate choice is non-Boolean.

### SHARED ENVIRONMENTAL CUES & DISTINCT FATES

#### A) Introduction: a common environment stimulates each fate

Given that all three diploid differentiation programs occur as nutrients become limiting, how does a cell choose between programs? It is possible that a small difference in the concentration of one or more nutrients under these conditions would result in passing a concentration threshold required for activation of a single fate choice. This would be an example of a cue/fate relationship that could be represented a Boolean logic since there are only two states relative to the threshold. This section addresses the question of whether the evidence allows for a Boolean relationship between cues and fate.

A summary of the relationships between environmental cues and differentiation pathways is shown in Fig. 3. Sporulation occurs under the specific condition of active respiration (NFCs being much higher than FCs), high pH, and depleted nitrogen and/or other nutrients (reviewed in 156,157). Quiescence, like sporulation, is triggered by the absence of at least one essential growth nutrient and alkaline pH, but (unlike sporulation) quiescence does not have a requirement for respiration (reviewed in 10,158. Finally, pseudohyphal differentiation occurs at intermediate-to-low nitrogen concentrations (reviewed in 135), can also be induced by other cues such as fusel alcohols, and also responds to other cues in some strain backgrounds (reviewed in 159).

#### B) Carbon source 

Yeast can metabolize many different carbon sources, but from the point of view of cell differentiation, there are two main types of carbon source. The first type is the FCs, in particular glucose, which is fermented through glycolysis to produce the second class - the NFCs. During late stages of growth, NFCs, particularly ethanol and acetate, are metabolized to carbon dioxide, and the balance between glucose and NFC during late stages of growth is a critical determinant of cell fate (Fig. 3).

##### 1) Carbon source, quiescence and sporulation.

 Glucose even in low concentrations effectively inhibits the initiation of meiosis in most laboratory strains. Presumably, sporulation has evolved such that it only initiates after the favored carbon source for growth, glucose, is fully metabolized to NFCs. For sporulation to initiate, a second cue besides the absence of glucose is the presence NFCs. Indeed, sporulation requires not only the energy provided by respiration but also the specific presence of a NFC 160, and the continued presence of NFCs is required even at late stages of sporulation 161. Thus, as the ratio of NFCs to glucose (or other FCs) increases at late stages of growth, the environment becomes increasingly optimized for sporulation (Fig. 3).

Quiescence is classically defined as occurring after the complete depletion of both NFCs and FCs 12. However, a core set of genes is induced during quiescence regardless of which nutrient (C, N or PO_4_) is limiting 162,163. Although quiescence can be induced in a range of environments, extracellular environment strongly influences the properties of Q cells. For example, metabolic and other biological properties of Q cells vary depending on which nutrient is limiting (reviewed in 10,164). These different properties suggest the existence of multiple types of Q cells depending on the presence or absence of particular environmental cues. For example, ethanol and acetic acid accelerate chronological ageing in Q cells 60,99,165,166.

##### 2) Carbon & phg.

A change in the ratio between NFCs and glucose likely regulates phg in all strain backgrounds, but the optimal NFC/glucose ratio may vary between backgrounds. For example, phg occurs efficiently in the Ʃ1278b background when grown on glucose medium, but is much more efficient in the SK1 background when grown on acetate medium 56,57. Several results may help to explain this difference. In the first place, even in Ʃ1278b, high concentrations of glucose (2%) inhibit haploid filamentous and invasive growth 167,168. In the second place, assays for phg in glucose medium generally involve cells dividing for many generations before N limitation triggers phg (termed the dimorphic switch) 169. Thus, at least some glucose has been converted to NFCs by the time phg initiates. In this respect, increased NFC/glucose ratio during late stages of growth may stimulate phg as well as sporulation; though the optimal ratio may be lower in Ʃ1278b than in SK1.

#### C) Nitrogen supply

##### 1) Nitrogen availability, growth and differentiation.

 It is tempting to neatly classify fate choice in yeast as driven by the presence or absence of two or three nutrients; yet, in nature yeast must make cell fate decisions across a range of different environments. The relative nitrogen availability from different sources illustrates this point. Yeast is capable of assimilating nitrogen from many different sources; however, the efficiency of utilizing nitrogen varies considerably depending on the source 170. For example, ammonium and glutamine are very good nitrogen sources for growth, whereas urea and tryptophan are poor sources. As discussed below, nitrogen "quality", besides affecting growth rate, also affects fate choice.

##### 2) Nitrogen and fate choice.

 In laboratory cultures, nitrogen is often the nutrient that becomes limiting during late stages of growth. In addition to directly regulating differentiation (see Section "Same signal paths – different fates"), nitrogen limitation prevents sufficient G1 cyclin from accumulating to activate the G1-to-S transition (START). The START transition blocks both sporulation and quiescence, both of which can only initiate from G1. Furthermore, cyclin expression inhibits both transcription of *IME1* 150,151,171 and nuclear localization of Ime1 172. Similarly, cyclin expression also blocks the establishment and maintenance of quiescence 79. Finally, once cells do enter quiescence, higher quality N sources accelerate ageing relative to lower quality sources 173.

An intermediate level of nitrogen is required both for phg and many phg-spectrum phenotypes 59. For example, *FLO11* is induced when low concentrations of nitrogen are present in the medium but repressed both at high nitrogen concentrations and when nitrogen is completely absent 174. Similarly, both *FLO11* induction and phg require intermediate G1 cyclin levels 128. Thus, phg requires both detecting and utilizing intermediate N concentrations 175,176,177.

In summary, nitrogen level/availability regulates cell fate, not just its presence or absence or its concentration relative to a single threshold. Thus, the role of nitrogen in regulating differentiation cannot be represented by a Boolean "1" vs. "0" relationship between this cue and cell fate.

#### D) The simplest of signals: pH 

##### 1) Extracellular pH fluctuates dramatically during growth.

 Changes in extracellular pH coincide with nutrient depletion and contribute to fate choice. Proliferation is much less sensitive to external pH than is differentiation - yeast grows efficiently through a broad pH range (pH 3-8) - in part because acid pumps provide a relatively uniform intracellular pH regardless of external pH 178. However, extracellular pH does change dramatically during both proliferation and differentiation. During fermentative growth, the secretion of organic acids such as acetate and pyruvate decreases external pH ≤ 4.0 166. In contrast, in media containing plentiful nitrogen and other nutrients, subsequent respiration of these organic acids during the diauxic shift converts these acids to C0_2_, which is either released as a gas or solubilized as bicarbonate. As a result, during the diauxic shift, extracellular pH increases to ≥ 8.0. In this respect, extracellular pH reflects the ratio of FCs to NFCs 179. Note that if the ratio of glucose to other essential nutrients is high enough in the chosen growth medium, then glucose will never be exhausted, and cells will not undergo either the diauxic shift or the second pH transition.

##### 2) Extracellular pH has complex effects on cell differentiation.

 Increased extracellular pH during late stages of growth stimulates quiescence 180. Likewise, when Q cells are exposed to low pH, their viability (chronological lifespan) is strongly diminished 166,181. Increased extracellular pH also activates sporulation, and this pH continues to rise as sporulation progresses 182,183. Finally, pH has varying effects on phg-spectrum of phenotypes. For example, flocculation is stimulated by acidic pH 58,184,185, whereas, invasive growth is stimulated by alkaline pH 186.

#### E) Is it really just all about the food? 

In natural environments, yeast must adapt to temperature fluctuations over the course of the day and the course of the year. In general, diploid differentiation is more sensitive to temperature than is proliferation. For example, most laboratory strains are unable to sporulate at moderately high temperatures (> 34°C) even though growth is still efficient at temperatures exceeding 37°C 187. Similarly, many clinical yeast isolates are able to grow as ovoid cells at much higher temperatures than as pseudohyphae 188,189. Finally, as cells enter quiescence, resistance to heat and other stress initially increases and then declines as cells age 190,191.

Another aspect of cellular environment is cell-cell contacts. The role of cell-cell contacts in cell-fate decisions is most clear for phg, where cell adhesion molecules like the flocculin, Flo11, and the mucin, Msb2, are required for many phg-spectrum phenotypes (reviewed in 192,193). Sporulation in minicolonies might also be regulated by cell-surface contacts, since only the pseudohyphae projecting from minicolonies, not cells in the core, are capable of sporulating 56.

#### F) Summary

Given that all three types of diploid differentiation occur in the nutrient-depleted environment characteristic of late stages of growth, it is not surprising that the environmental cues that regulate them are similar, but there are two points worth emphasizing. First, although each pathway occurs independently, there is no single combination of the presence or absence of cues that unambiguously specifies a single fate (Fig. 3). Second, the level or relative concentration of an environmental cue (e.g. ratio of NFCs to FCs or low, intermediate or high nitrogen) often correlates better with fate choice than simply the presence of that cue above some single threshold concentration. Both findings support the SEDF hypothesis and argue against a Boolean relationship between cues and fates.

### SAME SIGNAL PATHS - DIFFERENT FATES

#### A) Overview

It is striking that all three diploid differentiation pathways not only occur in similar nutrient environments but are also regulated by the same four major nutrient signal transduction pathways (PKA, TorC1, Snf1 and Rim101). As with master regulators and environmental cues, in the case of signaling pathways, one can imagine pathways in only one of two states, ON or OFF, relative to pathway targets including differentiation programs. Alternatively, there could be multiple or even continuous (graded) pathway activity levels. As one example, variable numbers of receptors could result in nearly continuous activity levels. The mechanisms and components of signal transduction have been discussed in several excellent recent reviews 3,61,194,195, so this section of the viewpoint focuses only on the role of these pathways in regulating fate choice and the possibility of a Boolean relationship between signal pathways and fate choice (Fig. 4).

#### B) Complex relationship between signal paths and nutrient signals

Study of most yeast nutrient signaling pathways initiated with a focus on the relationship between a single nutrient and a single pathway. For example, the TorC1 pathway is primarily activated by nitrogen, the Ras/PKA pathway is primarily activated by glucose and the Snf1 pathway is primarily repressed by glucose. However, it is now apparent that most nutrient-responsive pathways relay information about more than one type of nutrient. For example, multiple receptors responding to different classes of ligands can converge to regulate the same signaling pathway.

The Snf1 pathway exemplifies the ability of a single pathway to respond to diverse cues. Although this pathway was identified and characterized as active when glucose is absent, and is often referred to as the "glucose repression pathway", it also responds to other types of cellular stress (reviewed in 196). For example, in the absence of glucose, this pathway responds instead to nitrogen levels 197. Similarly, the TorC1 pathway is not only activated by abundant nitrogen, but also by glucose, by osmotic stress, and by other types of cellular stress 198. Finally, the PKA pathway, which is activated in high glucose through the Ras branch of the pathway, is also regulated through other branches that respond to carbon source, ammonium, amino acids and phosphate (reviewed in 199). In fact, even the Ras branch of the PKA pathway is not regulated solely by glucose but is sensitive to acetate levels 200 and may respond more generally to cytosolic pH 201,202.

#### C) PKA inhibits all three forms of differentiation 

PKA is active during rapid growth in FCs and has reduced activity in Q and sporulating cells. In yeast (and metazoans) PKA activates both the expression and activity of metabolic enzymes required for rapid growth. In addition, PKA represses genes required in non-proliferating, slow growing, and respiring cells (e.g. glycogen storage). Thus, PKA activity is low during all three types of cell differentiation.

PKA inhibits differentiation through both general mechanisms, which repress all three differentiation programs, and specific mechanisms, which repress only one (or two) of the three differentiation choices. One general mechanism for inhibition occurs when PKA phosphorylates Whi3 protein 203. Once phosphorylated, Whi3 releases *CLN3* mRNA, allowing progression of the cell cycle from G1 to S (START) and corresponding inhibition of quiescence and sporulation 203,204,205.

Another general mechanism by which PKA inhibits all three forms of differentiation is by phosphorylating and inactivating Rim15 kinase 206. Rim15 is required for the metabolic changes that accompany quiescence 207 and also activates the endosulfine, Igo1, which helps maintain mRNA populations during quiescence 208,209,210. Rim15 also activates the Msn2/Msn4 transcription factors, which directly activate stress response genes necessary for quiescence 86,211, and Rim15 is also required for the lifespan extension caused by calorie restriction 115,212. In addition to its role in quiescence, Rim15 is required for both the expression and activity of Ime1 213,214,215. Thus, Rim15 activates sporulation in part through different mechanisms from those it uses to activate quiescence 216. Finally, Rim15 is required for at least some phg-spectrum phenotypes, such as the formation of structured (lacey) colonies 131.

PKA also inhibits differentiation through regulators specific to just one or two differentiation fates. For example, PKA phosphorylates and activates the Sok2 transcriptional repressor 217,218. Active Sok2 directly represses *IME1* transcription, hence blocking sporulation 219 Sok2 also indirectly represses *FLO11* and other genes required for phg 220,221>. PKA also specifically inhibits spore morphogenesis by inhibiting Smk1 MAPK, which activates this late stage of sporulation 222. Finally, PKA also specifically inhibits both quiescence and phg by directly (and indirectly) inactivating Yak1 kinase 223,224. Yak1 is a key activator of proteins required for quiescence 225,226and phg 227. Thus, PKA represses all three forms of diploid cell differentiation through an array of general and specific mechanisms.

#### D) TorC1 pathway inhibits sporulation and quiescence/ageing, but activates phg

TorC1 is one of two Tor complexes in yeast with largely separate roles. When nutrients (especially N) are readily available, the TorC1 pathway activates cellular processes needed for rapid growth, such as protein translation. At the same time, this pathway represses processes induced during nutrient limitation such as utilization of poor nitrogen sources, autophagy, and the stress response.

Fully activated TorC1 directly represses both sporulation and entry into quiescence. TorC1 represses these programs through activating Sch9 kinase, which in turn inhibits Rim15 activity/nuclear localization 228. Thus, the TorC11 pathway acts in parallel to PKA in repressing Rim15 (reviewed in 229). TorC1 also specifically represses sporulation by preventing nuclear localization/activation of Ime1p 172. Interestingly, a low level of TorC1 is required for *IME1* expression; thus sporulation only initiates with moderate TorC1 activity 230. In addition, once cells become quiescent, activation of TorC1 diminishes their viability (i.e. promotes ageing) 89. Indeed, calorie restriction slows ageing by decreasing Sch9 activity 229,231,232.

In contrast to its role in inhibiting these other differentiation programs, TorC1 stimulates phg through a second branch of the TorC1 pathway, which activates the Tap42 phosphatase 233. Tap42 in turn stabilizes the Tec1 transcription factor, which binds *FLO11* and activates its transcription 234. Reflecting that optimal phg requires intermediate N concentrations (see Section "Shared environmental cues & distinct fates"), phg (like sporulation) also requires an intermediate level of TorC1 activity 174.

#### E) Snf1 kinase activates all three forms of differentiation

The Crabtree effect depends on the ability of extracellular glucose to repress enzymes required to metabolize other carbon sources. As levels of glucose and other nutrients decline during late stages of growth, repression of Snf1 kinase activity is released. Activated Snf1 in turn triggers the expression and/or activation of enzymes required for respiration and resistance to stress 235,236,237,238, and at the same time stimulates transcription of genes required for differentiation. For example, Snf1 activates *FLO11* transcription by phosphorylating Nrg1/Nrg2 transcription factors 167,239,240. Similarly, Snf1 is required for *IME2* transcription even in the absence of respiration 150. Finally, either hyper-activating or deleting Snf1 shortens chronological lifespan 115,241,242. Thus, maintaining viability during quiescence may require intermediate levels of Snf1 activity, similar to the intermediate TorC1 activity required for phg and sporulation. 

#### F) The Rim101 pathway activates both filamentous and meiotic fates

The Rim101 pathway is primarily activated by alkaline pH (reviewed in 243, and as cells undergo the diauxic shift and extracellular pH increases, this pathway stimulates both pseudohyphal differentiation and sporulation 186. For example, both *FLO11* induction and *IME1* induction require Rim101 182,244,245,246. Indeed, a *RIM101* polymorphism underlies many of the differences between Ʃ1278b and S288C transcriptomes during growth 247.

#### G) Other pathways and pathway interactions

The above signaling pathways are unlikely to be the only ones regulating differentiation. For example, the Rgt2 /glucose induction pathway, which is activated by glucose, represses Ime2 protein stability 248,249, the Hog1 MAPK pathway, which responds to high osmolarity, inhibits pseudohyphal differentiation 250,251, and the cell wall integrity (protein kinase C) pathway, which responds to cell-wall stress, is required both to maintain quiescent cells 252,253 and for pseudohyphal differentiation 254,255,256. Finally, the Hac1 (unfolded protein response) pathway, which responds to nitrogen starvation, mediates induction of both sporulation and phg 257. 

Not only does each of the nutrient-signaling pathways in yeast respond to multiple environmental cues, but also these pathways are not insulated from one another. Instead, the PKA, Snf1, TorC1 and other pathways are densely intertwined by virtue of shared components and shared targets (reviewed in 3,61,194). As just one example, the TorC1 pathway stimulates pseudohyphal differentiation in part by inhibiting Snf1 kinase 258, whereas the Rim101 and Snf1 pathways converge in both inhibiting Nrg1, a transcriptional activator of *FLO11* 259. Thus, nutrient controls on differentiation occur through an intertwined network of pathways.

#### H) Summary: signaling pathways and fate choice

As can be seen from Fig. 4, there is not a Boolean relationship between the four major nutrient signaling pathways and fate choice. In other words, fate choice is not determined by the ON or OFF state of one or more pathways (even relative to a threshold level of activity). Indeed, most of these pathways act in the same way on multiple fates. One exception is the TorC1 pathway, which activates phg while interfering with the other two pathways. However, even TorC1 does not act in a strictly Boolean manner, since several lines of evidence suggest an intermediate level of TorC1 activity, rather than either the fully active or fully inactive state is what activates both phg and sporulation. More generally, because these pathways regulate initiation of all 3 fates, the level of activation of each pathway, rather than a Boolean state, determines fate choice. Further work is required to ask if the activity level of one pathway relative to another contributes to fate choice. 

### FLEXIBILITY & STABILITY IN DIFFERENTIATION CHOICE

#### A) Fate choice stability

Another aspect of the relationship between fate choice and environment is the stability and reversibility of this choice. At one level, all three types of differentiated cells are reversible, in the sense that if sufficient nutrients are restored, pseudohyphae, Q cells and asci all re-enter standard cell proliferation. Furthermore, under some circumstances yeast can switch from one fate to another without intervening growth as undifferentiated cells. In this section, the SEDF hypothesis will be considered in the context of the stability /reversibility of fate choice.

#### B) Epigenetic mechanisms stabilize cell fate

Each fate is in part stabilized by genome-wide epigenetic changes. For example, although pseudohyphae can produce ovoid cells when restored to a very nutrient-rich environment, phg is relatively stable to small changes in environment 59,159. One mechanism for this stability is that the *FLO11* promoter is bi-stable, i.e. it stays in either the ON or OFF state for many cell divisions before switching, and this switch depends on chromatin structure and in particular on histone modifying enzymes 260,261 and long non-coding RNA 262,263,264. *FLO11* expression is also stabilized by a prion-like form of the transcription factor, Mot3. Soluble Mot3 represses *FL011* transcript, and this repression is released when Mot3 forms heritable prions 265. 

Both quiescence and sporulation are also characterized by chromatin modifications. For example, genome-wide alterations in histone modification accompany both progression through sporulation 266,267, and transcriptional inactivation after sporulation is complete 268. Similarly, quiescence is characterized by both global changes in histone modification 78,269, and positioning of RNAPII upstream of many genes poised for induction when nutrients are restored 270. 

Once cells initiate sporulation, it is only during the early stages of this program that the meiotic fate is directly reversible. Reversibility in this context means that cells in early stages of meiosis can directly re-enter proliferation if resupplied with nutrients ("return to growth"). The loss of this reversibility, termed "commitment to meiosis", may also have its basis in epigenetics. More specifically, when cells in these early stages are resupplied with growth nutrients, they exit sporulation and resume cell division; however, at about the time of the first meiotic division, cells become irreversibly committed to meiosis, meaning that they complete sporulation even when resupplied with growth nutrients 271,272. Commitment to meiosis may involve a positive feedback loop regulating transcription of *NDT80* 273,274, and as with *FLO11*, the silenced state of *NDT80* requires a histone deacetylase 222,275. Commitment to meiosis may also involve a second epigenetic mechanism - Rim4 forms an amyloid-like protein that binds transcripts during sporulation to delay their translation until late stages of the program 276, and it may be that binding of the amyloid to these transcripts also protects them from degradation after nutrients are resupplied 277.

#### C) When yeast change their fate

In some circumstances, cells can switch from one differentiation fate to another. For example, the SK1 laboratory strain background can switch from meiotic development to pseudohyphal development in response to a changing environment 143. Even committed meiotic cells, if arrested at late stages of meiosis, can eventually re-enter the cell division cycle as pseudohyphae 278,279. 

Even without shifting media or environment, biofilm-like communities of the SK1 strain background form pseudohyphae at their surface that subsequently undergo a further differentiation to form linear asci 56,57. Similarly, as noted in the Section "Overlapping regulators but distinct fates", the shared expression pattern between quiescence and sporulation suggest that cells first enter quiescence and then proceed to sporulation, again without being transferred to a new environment. These several lines of evidence indicate that each of three fates can be temporally coordinated with at least one other fate. 

#### D) Summary

A variety of epigenetic mechanisms likely provide stability to yeast differentiation fates, but it is interesting that both phg and sporulation fates are stabilized by amyloid/prion proteins as well as by chromatin modification. Despite these mechanisms, differentiating cells can return to undifferentiated proliferation in response to environmental changes - in some cases even before differentiation is complete. Moreover, yeast can switch from one differentiation pathway to another even in the absence of dramatic environmental changes. The reversibility and flexibility of fate choice in yeast is another argument that distinct fates can occur in a similar environment (SEDF), and against a Boolean mechanism of fate choice.

### SHARED COMMUNITIES - COORDINATED FATES

#### A) Introduction

Yeast grow in nature as communities, not as suspended cultures. Given that different fates respond to the same cues and regulators and that these fates sometimes interconvert, one might expect that in the same population and community, some cells might adopt one fate and some cells another. In fact, as described below, this is exactly what happens both in communities and suspended cultures. Thus, the question of whether different fates are triggered by discrete cues and regulators can be asked in the context of yeast populations. As described below, communities that contain cells adapting different fates are particularly relevant to the SEDF hypothesis.

#### B) Fate partitioning within populations

##### 1) Fate choice in cultures.

 Before discussing fate choice within communities, it is worth considering fate choice in suspended cultures. Microenvironment can vary between different regions of a community, whereas suspended cultures provide the opportunity to measure fate choice within a population with both uniform genotype and uniform environment. One aspect revealed in suspended cultures is the probabilistic nature of fate choice. That is, depending on the conditions of the cultures, a given fraction of the culture adapts a particular fate. As described below, the probabilistic aspect of fate choice is clearest in cultures in which the population in the culture divides into two alternative differentiation fates.

Sporulation is particularly useful in comparing cell fates. For most laboratory strains suspended sporulation cultures (e.g. media containing only 2% potassium acetate) contains both sporulated and unsporulated cells. The unsporulated sub-population likely corresponds to Q cells, since this subpopulation can be further subdivided, by cytological markers into viable cells, those undergoing PCD, and those already dead 280. 

Similar to diploid cultures, haploid suspended cultures grown for extended periods form at least two populations of cells, designated Q and non-quiescent (NQ) cells. Q cells differ from NQ cells in many properties, including density, stress resistance, lifespan, transcriptome and proteome 152,281,282. 

##### 2) Examples of the spatial organization of cell fate within communities.

 As they proliferate, yeast naturally form tightly packed multi-cellular communities such as colonies and biofilms (reviewed in 283,284). Strikingly, differentiation is not homogenously distributed throughout the community, but instead occurs in specific regions. Below are four examples of yeast communities in which cell fate is spatially organized in communities either with respect to undifferentiated cells or with respect to cells adapting a different fate. 

a) Sporulation patterns in diploid colonies: Patterns of sporulated cells in colonies are easily visible in embedded sections of colonies 182,285. Specifically, asci are found throughout the top half of mature colonies and also in a thin layer of cells near the agar surface. In contrast, asci are almost completely absent throughout the broad central layer of the colony. Furthermore, the boundaries between sporulating and non-sporulating cells are sharply defined, and this same pattern is observed in a range of laboratory and natural yeast strains. Indeed, strains newly isolated from the wild form this pattern on both FCs and NFCs and on both synthetic and rich media 25.

The underlying layer of unsporulated cells can be considered a type of quiescent cell, and are termed "feeder cells" because they remain viable for many days, increase in permeability and stimulate sporulation in the overlying cell layer 74. As discussed in the Section "Cell-cell signals in communities determine cell fate", this stimulation is thought to occur because feeder cells provide nutrients to overlying cells, with the increased permeability dependent on induction of the CWI pathway. 

b) Upper (U) and Lower (L) cell layers in haploid colonies: Haploid colonies form a similar pattern of differentiation as the diploid colonies described above, although of course they do not sporulate. Haploid colonies form two sharply defined layers of cells- termed U and L cells 286,287. U cells differ from L cells with respect to size, morphology, metabolism, gene expression, and viability. 

c) Sexual reproduction on the fringe: Both phg and sporulation occur specifically at the edge of some communities. For example, small colonies forming on agar media limiting for nitrogen (microcolonies) form a core of (typically-shaped) ovoid yeast with only the outer surface of the colony containing chains of pseudohyphae projecting from the colony 59. Similarly, minicolonies, biofilm-like communities growing on plastic surfaces submerged in medium, switch from typical yeast divisions at the early stages of cell growth to phg at the periphery as the growth begins to slow 56. Biofilms formed by pathogenic yeast such as *C. albicans* form a similar pattern in that the underlying layer of cells is comprised of ovoid cells, whereas the top layer consists of hyphae 288,289. 

As described above, the SK1 strain background both sporulates and undergoes phg very efficiently. As mentioned in the Section "Flexibility & stability in differentiation choice", the pseudohyphae at the periphery of SK1 microcolonies and minicolonies subsequently sporulate to form linear 2-, 3, and 4-spore asci 56,57. In minicolonies, the timing of the transition from phg to sporulation has been followed by time-lapse microscopy. In these communities sporulation occurs synchronously around the colony edge but never in the interior of the colony. Furthermore, if phg is blocked, sporulation is also prevented 56. Thus in these communities, phg and meiotic differentiation are sequential, rather than alternative, fates.

d) PCD patterning in colonies: Like sporulation and pseudohyphal differentiation, PCD does not occur uniformly throughout communities. In spot colonies growing on a NFC for 1-3 weeks, apoptosis occurs mostly in the cells that form the core of the colony. In contrast, cells on the rim and outer surface of the colony maintain high viability 290. It is possible that the high levels of PCD in the core increase the survival or growth among cells at the rim of the same colony, perhaps by providing scarce resources from the PCD region to the viable region 291. 

##### 3) Sharp boundaries as a common characteristic of community patterning. 

A striking characteristic of pattern formation in both colonies and biofilms is the sharply defined boundary surrounding regions of differentiation (reviewed in 284). These boundaries can be visualized not only with respect to cytological markers like apoptosis, spore formation, and phg as described above, but also by patterns of gene expression 74,182,286,287. Indeed, on one side of a boundary, cells differentiate very efficiently to one fate, whereas neighboring cells in contact with these cells but on the other side of the boundary differentiate equally efficiently but to a different fate.

#### C) Summary

The SEDF hypothesis, that cells can adopt different fates in similar environments, is strongly supported by the sharp boundaries observed between neighboring regions of a yeast community. That is, cells on either side of a boundary share roughly the same environment but adopt distinct fates. In contrast, these boundaries are not easy to reconcile with a Boolean model of fate choice. In the next section we discuss one mechanism allowing SEDF - cell-to-cell signals.

### CELL-CELL SIGNALS IN COMMUNITIES DETERMINE CELL FATE

#### A) Introduction: role of cell-cell signals in cell fate and patterning

As described above, pseudohyphal differentiation, quiescence/aging and sporulation occur under similar conditions (SEDF), are regulated by many of the same signaling pathways and master regulators, and often occur together in the same community. Taken together these results argue against a Boolean relationship between input and cell fate. Given this, how do neighboring cells along a boundary efficiently adopt different fates, and how has yeast evolved to make the correct fate choice across the wide spectrum of environments found in nature? As discussed below, one possible answer to both of these questions is cell-to-cell signaling. 

Signals between individual yeast cells within communities are typically small diffusible molecules (reviewed in 284). Below I consider two broad classes of cell-cell signals in yeast: 1) "enlistment" signals, which are produced by differentiating cells within one region of a community to stimulate cells within the same region to adopt the same fate, and 2) "diplomatic signals", which are produced by differentiating cells in one region of the community to influence cells in an adjoining region to adopt a different fate.

#### B) Enlistment signals - intercellular feedback stimulates differentiation & patterning

Enlistment signals, by reinforcing the same fate choice in neighboring cells, contribute to the spatial organization of communities such that many of the cells within one region of the community adopt the same fate. 

##### 1) Alkali signals and sporulation patterning.

 As described in the Section "Shared environmental cues & distinct fates" above, respiration of organic acids during late stages of growth and during sporulation leads to an increase in extracellular pH, which in turn stimulates sporulation in other cells of the same population. In colonies, after a narrow layer of cells near the center initiates sporulation, the resulting alkalization progressively drives expansion of the sporulating region upward to eventually include the entire top half of the colony 182. This wave of sporulation depends on the Rim101 pathway both to sense and to produce alkali - suggesting an intercellular positive feedback loop involving pH signals. 

##### 2) Aromatic alcohols and pseudohyphal patterning.

 Regulation of the dimorphic switch in microcolonies also involves metabolites and an intercellular positive feedback loop 292. In the case of phg, these signals include the aromatic alcohols phenylethanol and tryptophol. These alcohols are produced and secreted during phg, and in turn stimulate phg in surrounding cells. Moreover, extracellular tryptophol induces further synthesis of tryptophol within pseudohyphal cells, which further amplifies the feedback loop 292. 

##### 3) Role of ammonium in cycles of proliferation & quiescence.

 Another example of a cell-cell signal that operates in yeast communities is the ammonium produced by haploid colonies after ≥ 14 d of growth. Cells on the surface of these aged colonies produce ammonium to much higher levels than cells in the core of these colonies 290,293. At the same time, surface cells induce expression of the *ATO1* ammonium exporter 294. Ammonium contributes to the survival of surface cells and to a new cycle of proliferation at the edge of the colony 295. Because surface cells both produce and respond to ammonium, ammonium is another example of enlistment signals acting through an intercellular positive feedback loop. Indeed, ammonium can diffuse from one colony to its neighbors, synchronizing these colonies with respect to their growth/quiescence cycles 296. 

In summary, enlistment signals such as alkali, aromatic alcohols or ammonium may all act through a common mechanism - an intercellular positive-feedback loop. The role of these signals varies; of the three signals discussed above, one activates sporulation, another activates phg, and a third activates proliferation. These feedback loops amplify relatively small differences in nutrient microenvironment to generate larger environmental differences. In this respect, intercellular positive feedback loops contribute to community pattern formation by localizing cell-cell signals to specific regions of a community. 

#### C) Diplomatic signals - crossing boundaries to influence behavior

##### 1) The Rlm1 paradigm and the DPEB hypothesis.

**Figure 5:**
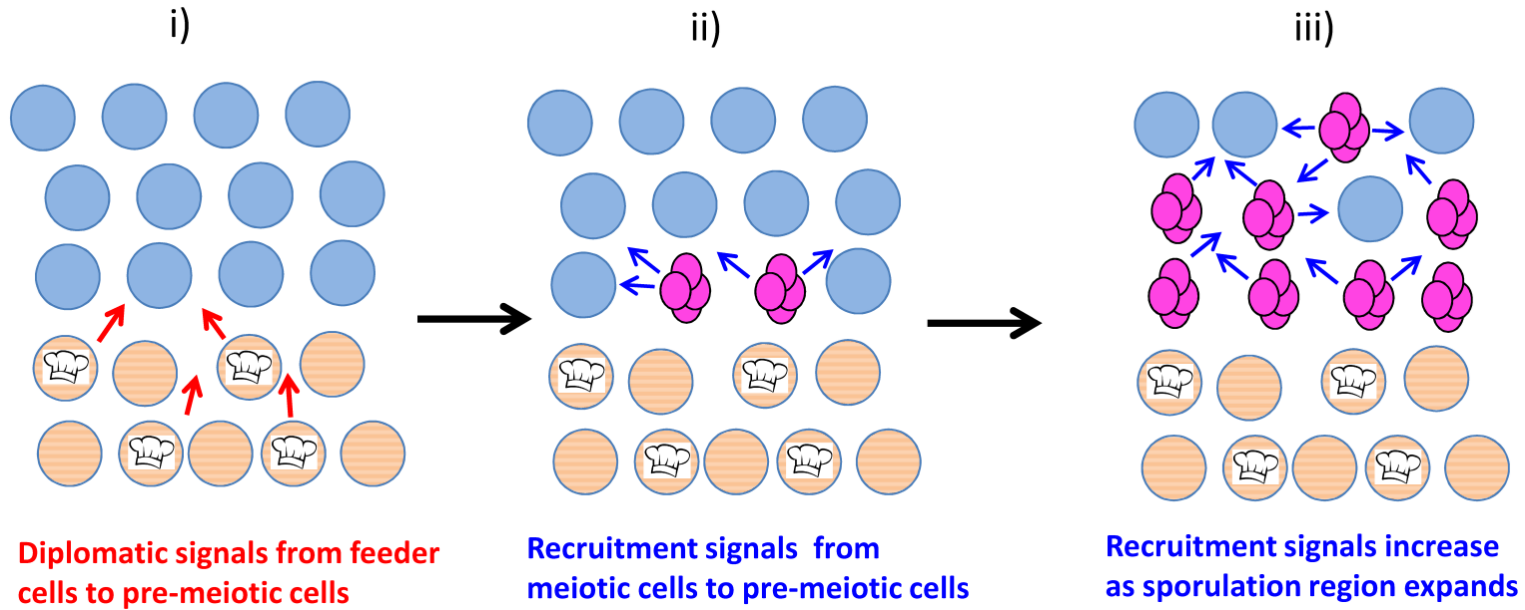
FIGURE 5: Recruitment and diplomatic signals combine to partition colonies into regions adopting different fates. **(i)** After growth of colony is complete, cells in the underlying cell layer (beige) differentiate into a type of quiescent cells termed "feeder cells". Feeder cells (designated by a chef’s hat) remain viable but have increased permeability, allowing them to provide nutrients and/or other signals (red arrows) to the overlying layer of cells (light blue). **(ii)** These "diplomatic signals" between regions of the yeast community promote sporulation in upper layer cells near the border (tetrad asci are shown in magenta). As these first cells sporulate, continued respiration of acetate in these cells causes alkalization of the microenvironment. The alkali produced by sporulating cells is a "recruitment signal" (blue arrows) to trigger sporulation in surrounding cells in this layer.**(iii)** This intercellular positive feedback loop involving sporulation and alkalization results in an upward wave of sporulation eventually including the entire upper region of the colony.

 In contrast to enlistment signals, diplomatic signals occur between community regions adopting different fates. An example of this second type of cell-to-cell signal occurs in sporulating colonies, where activation of the Rlm1 transcription factor in feeder cells stimulates sporulation in an overlying layer of cells through a cell non-autonomous mechanism 74. Thus, sporulating colonies contain both recruitment and diplomatic signals (Fig. 5). 

This type of diplomatic signal may allow yeast colonies to sporulate in a wider range of environments than possible for individual cells, a hypothesis termed, "differential partitioning provides environmental buffering" or DPEB 73,74. According to this hypothesis, under optimal sporulation conditions, the colony is partitioned such that there are relatively few cells allocated to the feeder cell fate. In contrast, under suboptimal sporulation conditions, a greater portion of the colony is given over to the feeder cell fate, and overall colony sporulation is highly dependent on the nutrients and/or signals provided by these feeder cells. Thus, increasing the proportion of feeder cells buffers sporulation efficiency in suboptimal environments. Differential partitioning is a form of phenotypic plasticity within communities that is related in some ways to task allocation in social organisms such as ant, termite and bee species 297,298.

##### 2) Last gasp diplomatic signal.

 Another example of signals between different regions of the same colony may occur in the haploid colonies undergoing age-triggered PCD described in the Section "Shared communities – coordinated fates". In these colonies, PCD in the colony core is postulated to provide nutrients to continued cell proliferation at the colony’s rim 290. Because intracellular ROS levels, including H_2_O_2_, which is relatively stable in an extracellular environment, rise in core cells even at relatively early stages of development 299,300, it is possible that peroxide is a diplomatic signal. Thus diplomatic signals, either from feeder cells or PCD cells, may contribute to forming and maintaining the sharp boundaries between regions undergoing alternative fates.

#### D) Summary: Boolean logic and cell-cell signals

In several types of yeast communities, closely neighboring cells on either side of a boundary adopt alternative fates consistent with the SEDF hypothesis but not Boolean models. For yeast to adapt distinct fates in the very similar nutrient environments on either side of a boundary, cell-cell signals are likely essential. The cell-cell signals so far identified may represent only a fraction of the signals operating in yeast communities. 

Enlistment and diplomatic cell-cell signals cooperate to reinforce small differences in the nutrient microenvironment and are likely important to both establish and maintain boundaries. For example, enlistment signals within one region of a community can amplify differences via intercellular positive feedback loops; whereas, diplomatic signals between neighboring regions can help enforce the sharp boundary between regions. Similar mechanisms are critical to forming boundaries between tissues during metazoan development 301,302. Finally, cell-cell signals can provide biological function not available to individual cells, such as buffering the efficiency of a differentiation program against unfavorable environments (DPEB).

### ORIGINS OF COMMUNICATION & OF MULTICELLULARITY 

#### A) Looking backwards: ancient communities & the birth of communication

What are the broader implications of the non-Boolean SEDF hypothesis and the biological function of pattern formation in yeast communities? Organized patterns within microbial communities date back to the earliest life on earth. For example, stromatolite fossils from billions (109) of years ago provide evidence of organization within cyanobacteria communities (reviewed in 303,304). 

The selection pressure for communication to evolve in microbes can be placed in the context of the more complicated forms of communication that evolved in complex organisms. In this respect, it is relevant that some of the most successful metazoan species on earth as judged by total biomass (e.g. humans, termites and ants), are those with highly developed modes of communication. It has been suggested that the "simple multicellularity" that exists in microbial communities differs from the "complex multicellularity" characteristic of plants and animals because of two properties present only in complex organisms: 1) many cells in complex systems do not make contact with the external environment, and 2) high levels of cell-cell communication 305,306. However, a close look at the biology of yeast communities calls these distinctions into question.

#### B) The advantages of communication & community

Cell signaling between yeast cells hints at the advantages gained from the evolution of communication. In this respect, most of the signaling molecules discussed above share two common characteristics. First, they are important in regulating cell differentiation. Second, they act within the context of a community of yeast, often to partition this community into different regions adopting distinct cell fates. 

One of the main advantages to yeast of growing within a community may lie in the ability of a community to partition into regions adopting different fates; these regions may cooperate to allow greater biological function than is possible for individual cells. For example, limiting pseudohyphal and meiotic differentiation to the edge of minicolonies and microcolonies maximizes the ability of spores to disperse from the colony 56,57. As a second example, in colonies the underlying layer of differentiated feeder cells presumably provides nutrients necessary for sporulation in the overlying cell layer, which again is more optimal for spore dispersal. Finally, PCD in the core of a haploid colony may provide the nutrients that allow proliferation at the colony’s rim. Note that because natural yeast communities are largely clonal, the survival of the genotype depends on the overall survival of the colony, rather than competition between *S. cerevisiae* genotypes in the same community. To extrapolate further, the likely clonal nature of early microbial communities may have led not only to the evolution of the first cell-cell signals on earth but also to the first pattern formation. 

#### C) Model for co-evolution of signaling and multicellularity

##### 1) From response to communication.

 The ability of organisms to respond to their environment is expected even in the earliest life forms. Cell-to-cell signals in modern yeast may yield insight into these ancient signals. In particular, many yeast cell-cell signals are simply metabolites produced by nearby organisms such as alkali generated during respiration or ammonium produced by Q cells. Thus, the earliest form of communication between organisms may have evolved as metabolic byproducts coordinating growth with differentiation. 

##### 2) From communication to organization.

 Cell-cell signals in yeast not only regulate cell-fate, they also organize communities such that different regions adopt different fates. By analogy, the response to primordial cell-cell signals may have evolved such that variation in signal concentration across a community contributed to the organization of this community and hence increased fitness.

##### 3) From organization to multicellularity.

 The patterning of cell types in microbial communities may presage cell type patterning within complex multicellular organisms. Indeed, it is conceivable that the earliest multicellular organisms evolved from microbial species with relatively sophisticated cell type patterns. Conversely, patterning in modern microorganisms may provide a useful model for some of the fundamental principles guiding pattern formation in all organisms.

### CONCLUSIONS

#### A) Biology ain’t always Boolean

Similar environmental cues promote each of three alternative differentiation fates (SEDF), and these cues act through many of the same signal transduction pathways and master regulators to control fate choice. Thus, a Boolean representation tracking the presence or absence of a given cue or cues (or a discrete threshold) is not sufficient to describe the relationship between environmental cues and fate choice. Similarly, the relationship between fate choice and signal pathway/master regulator activity also cannot be accurately represented by Boolean logic. 

#### B) Graded and specialized responses 

So how is fate chosen? In the first place, it is likely that signal transduction pathways are not simply ON or OFF, but have a graded range of activities. By this view, the relative level of activity of signaling pathways determines cells fate as is seen for the TorC1 pathway. For example, the number of activated receptors may vary depending on the concentration of ligand. Indeed, the same signaling pathway might regulate different targets depending on its level of activity. In any case, the activity of a given signal transduction pathway likely also depends on interactions with other signaling pathways.

#### C) Cell-cell signals reinforce differences and provide flexibility

A second aspect to the choice of differentiation fates is provided by cell-cell signals. Relatively small differences in the microenvironment around cells can be reinforced or amplified by both recruitment and diplomatic cell-cell signals. These signals organize yeast communities into cooperative assemblies such that programs such as differentiation or proliferation occur more efficiently than is possible for individual cells. Of particular note is the ability of communities to adjust the allocation of cells to different fates within the community depending on the environment (DPEB). In microorganisms as in more complex organisms, cell-to-cell signals are fundamental to life and may be nearly as ancient.

## References

[1] Veliz-Cuba A, Stigler B (2011). Boolean models can explain bistability in the lac
operon.. J Comput Biol.

[2] Botstein D, Fink GR (2011). Yeast: an experimental organism for 21st Century
biology.. Genetics.

[3] Zaman S, Lippman SI, Zhao X, Broach JR (2008). How Saccharomyces responds to nutrients.. Annu Rev Genet.

[4] Schneper L, Duvel K, Broach JR (2004). Sense and sensibility: nutritional response and signal
integration in yeast.. Curr Opin Microbiol.

[5] Neiman AM (2011). Sporulation in the budding yeast Saccharomyces
cerevisiae.. Genetics.

[6] Kupiec M, Byers B, Esposito RE, Mitchell AP (1997). Meiosis and sporulation in Saccharomyces
cerevisiae.. In: Pringle JR, Broach JR, Jones EW, editors. The molecular and cellular
biology of the yeast Saccharomyces: Cell cycle and cell biology..

[7] Dickinson JR (2008). Filament formation in Saccharomyces cerevisiae-a
review.. Folia Microbiol (Praha).

[8] Gagiano M, Bauer FF, Pretorius IS (2002). The sensing of nutritional status and the relationship to
filamentous growth in Saccharomyces cerevisiae.. FEMS Yeast Res.

[9] Gancedo JM (2001). Control of pseudohyphae formation in Saccharomyces
cerevisiae.. FEMS Microbiol Rev.

[10] Palkova Z, Wilkinson D, Vachova L (2014). Aging and differentiation in yeast populations: elders with
different properties and functions.. FEMS Yeast Res.

[11] De Virgilio C (2012). The essence of yeast quiescence.. FEMS Microbiol Rev.

[12] Gray JV, Petsko GA, Johnston GC, Ringe D, Singer RA, Werner-Washburne M (2004). "Sleeping beauty": quiescence in Saccharomyces
cerevisiae.. Microbiology and molecular biology reviews : MMBR.

[13] Liti G, Carter DM, Moses AM, Warringer J, Parts L, James SA, Davey RP, Roberts IN, Burt A, Koufopanou V, Tsai IJ, Bergman CM, Bensasson D, O'Kelly MJ, van Oudenaarden A, Barton DB, Bailes E, Nguyen AN, Jones M, Quail MA, Goodhead I, Sims S, Smith F, Blomberg A, Durbin R, Louis EJ (2009). Population genomics of domestic and wild yeasts.. Nature.

[14] Naumov GI, Naumova ES, Sniegowski PD (1998). Saccharomyces paradoxus and Saccharomyces cerevisiae are
associated with exudates of North American oaks.. Can J Microbiol.

[15] Wang QM, Liu WQ, Liti G, Wang SA, Bai FY (2012). Surprisingly diverged populations of Saccharomyces cerevisiae in
natural environments remote from human activity.. Mol Ecol.

[16] Aa E, Townsend JP, Adams RI, Nielsen KM, Taylor JW (2006). Population structure and gene evolution in Saccharomyces
cerevisiae.. FEMS Yeast Res.

[17] Fay JC, Benavides JA (2005). Evidence for domesticated and wild populations of Saccharomyces
cerevisiae.. PLoS Genet.

[18] Hose J, Yong CM, Sardi M, Wang Z, Newton MA, Gasch AP (2015). Dosage compensation can buffer copy-number variation in wild
yeast.. Elife.

[19] Cubillos FA, Louis EJ, Liti G (2009). Generation of a large set of genetically tractable haploid and
diploid Saccharomyces strains.. FEMS Yeast Res.

[20] Hope EA, Dunham MJ (2014). Ploidy-regulated variation in biofilm-related phenotypes in
natural isolates of Saccharomyces cerevisiae.. G3 (Bethesda).

[21] Albertin W, Marullo P (2012). Polyploidy in fungi: evolution after whole-genome
duplication.. Proceedings Biological sciences / The Royal Society.

[22] Sicard D, Legras JL (2011). Bread, beer and wine: yeast domestication in the Saccharomyces
sensu stricto complex.. C R Biol.

[23] Storchova Z (2014). Ploidy changes and genome stability in yeast.. Yeast.

[24] Gerke JP, Chen CT, Cohen BA (2006). Natural isolates of Saccharomyces cerevisiae display complex
genetic variation in sporulation efficiency.. Genetics.

[25] Piccirillo S, Honigberg SM (2010). Sporulation patterning and invasive growth in wild and
domesticated yeast colonies.. Res Microbiol.

[26] Sniegowski PD, Dombrowski PG, Fingerman E (2002). Saccharomyces cerevisiae and Saccharomyces paradoxus coexist in a
natural woodland site in North America and display different levels of
reproductive isolation from European conspecifics.. FEMS Yeast Res.

[27] Zorgo E, Chwialkowska K, Gjuvsland AB, Garre E, Sunnerhagen P, Liti G, Blomberg A, Omholt SW, Warringer J (2013). Ancient evolutionary trade-offs between yeast ploidy
states.. PLoS Genet.

[28] Sherwood RK, Scaduto CM, Torres SE, Bennett RJ (2014). Convergent evolution of a fused sexual cycle promotes the haploid
lifestyle.. Nature.

[29] Merlini L, Dudin O, Martin SG (2013). Mate and fuse: how yeast cells do it.. Open Biol.

[30] Ruderfer DM, Pratt SC, Seidel HS, Kruglyak L (2006). Population genomic analysis of outcrossing and recombination in
yeast.. Nat Genet.

[31] Diezmann S, Dietrich FS (2009). Saccharomyces cerevisiae: population divergence and resistance to
oxidative stress in clinical, domesticated and wild
isolates.. PLoS One.

[32] Magwene PM, Kayikci O, Granek JA, Reininga JM, Scholl Z, Murray D (2011). Outcrossing, mitotic recombination, and life-history trade-offs
shape genome evolution in Saccharomyces cerevisiae.. Proceedings of the National Academy of Sciences of the United States of
America.

[33] Stefanini I, Dapporto L, Berna L, Polsinelli M, Turillazzi S, Cavalieri D (2016). Social wasps are a Saccharomyces mating nest.. Proc Natl Acad Sci U S A.

[34] Knop M (2006). Evolution of the hemiascomycete yeasts: on life styles and the
importance of inbreeding.. Bioessays.

[35] Mitchell AP, Herskowitz I (1986). Activation of meiosis and sporulation by repression of the RME1
product in yeast.. Nature.

[36] Wagstaff JE, Klapholz S, Esposito RE (1982). Meiosis in haploid yeast.. Proc Natl Acad Sci U S A.

[37] van Dijken JP, Weusthuis RA, Pronk JT (1993). Kinetics of growth and sugar consumption in
yeasts.. Antonie Van Leeuwenhoek.

[38] Kim JH, Roy A, Jouandot 2nd D, Cho KH (2013). The glucose signaling network in yeast.. Biochim Biophys Acta.

[39] Dashko S, Zhou N, Compagno C, Piskur J (2014). Why, when, and how did yeast evolve alcoholic
fermentation?. FEMS Yeast Res.

[40] Goddard MR, Greig D (2015). Saccharomyces cerevisiae: a nomadic yeast with no
niche?. FEMS Yeast Res.

[41] Paczia N, Nilgen A, Lehmann T, Gatgens J, Wiechert W, Noack S (2012). Extensive exometabolome analysis reveals extended overflow
metabolism in various microorganisms.. Microb Cell Fact.

[42] Zampar GG, Kummel A, Ewald J, Jol S, Niebel B, Picotti P, Aebersold R, Sauer U, Zamboni N, Heinemann M (2013). Temporal system-level organization of the switch from glycolytic
to gluconeogenic operation in yeast.. Mol Syst Biol.

[43] Schuller HJ (2003). Transcriptional control of nonfermentative metabolism in the
yeast Saccharomyces cerevisiae.. Curr Genet.

[44] Kumar R, Dhali S, Srikanth R, Ghosh SK, Srivastava S (2014). Comparative proteomics of mitosis and meiosis in Saccharomyces
cerevisiae.. Journal of proteomics.

[45] Grassl J, Scaife C, Polden J, Daly CN, Iacovella MG, Dunn MJ, Clyne RK (2010). Analysis of the budding yeast pH 4-7 proteome in
meiosis.. Proteomics.

[46] Walther T, Letisse F, Peyriga L, Alkim C, Liu Y, Lardenois A, Martin-Yken H, Portais JC, Primig M, Francois J (2014). Developmental stage dependent metabolic regulation during meiotic
differentiation in budding yeast.. BMC biology.

[47] Ray D, Ye P (2013). Characterization of the metabolic requirements in yeast
meiosis.. PLoS One.

[48] Mortimer RK, Johnston JR (1986). Genealogy of principal strains of the yeast genetic stock
center.. Genetics.

[49] Deutschbauer AM, Davis RW (2005). Quantitative trait loci mapped to single-nucleotide resolution in
yeast.. Nat Genet.

[50] Ben-Ari G, Zenvirth D, Sherman A, David L, Klutstein M, Lavi U, Hillel J, Simchen G (2006). Four linked genes participate in controlling sporulation
efficiency in budding yeast.. PLoS Genet.

[51] Casalone E, Barberio C, Cappellini L, Polsinelli M (2005). Characterization of Saccharomyces cerevisiae natural populations
for pseudohyphal growth and colony morphology.. Res Microbiol.

[52] Song Q, Johnson C, Wilson TE, Kumar A (2014). Pooled segregant sequencing reveals genetic determinants of yeast
pseudohyphal growth.. PLoS Genet.

[53] Liu H, Styles CA, Fink GR (1996). Saccharomyces cerevisiae S288C has a mutation in FLO8, a gene
required for filamentous growth.. Genetics.

[54] Jubany S, Tomasco I, Ponce de Leon I, Medina K, Carrau F, Arrambide N, Naya H, Gaggero C (2008). Toward a global database for the molecular typing of
Saccharomyces cerevisiae strains.. FEMS Yeast Res.

[55] Van Mulders SE, Christianen E, Saerens SM, Daenen L, Verbelen PJ, Willaert R, Verstrepen KJ, Delvaux FR (2009). Phenotypic diversity of Flo protein family-mediated adhesion in
Saccharomyces cerevisiae.. FEMS Yeast Res.

[56] White MG, Piccirillo S, Dusevich V, Law DJ, Kapros T, Honigberg SM (2011). Flo11p adhesin required for meiotic differentiation in
Saccharomyces cerevisiae minicolonies grown on plastic
surfaces.. FEMS Yeast Res.

[57] Strudwick N, Brown M, Parmar VM, Schroder M (2010). Ime1 and Ime2 are required for pseudohyphal growth of
Saccharomyces cerevisiae on nonfermentable carbon sources.. Mol Cell Biol.

[58] Bayly JC, Douglas LM, Pretorius IS, Bauer FF, Dranginis AM (2005). Characteristics of Flo11-dependent flocculation in Saccharomyces
cerevisiae.. FEMS Yeast Res.

[59] Gimeno CJ, Ljungdahl PO, Styles CA, Fink GR (1992). Unipolar cell divisions in the yeast S. cerevisiae lead to
filamentous growth: regulation by starvation and RAS.. Cell.

[60] Fabrizio P, Gattazzo C, Battistella L, Wei M, Cheng C, McGrew K, Longo VD (2005). Sir2 blocks extreme life-span extension.. Cell.

[61] Engelberg D, Perlman R, Levitzki A (2014). Transmembrane signaling in Saccharomyces cerevisiae as a model
for signaling in metazoans: state of the art after 25 years.. Cellular signalling.

[62] Coluccio AE, Rodriguez RK, Kernan MJ, Neiman AM (2008). The yeast spore wall enables spores to survive passage through
the digestive tract of Drosophila.. PLoS ONE.

[63] Granot D, Snyder M (1991). Glucose induces cAMP-independent growth-related changes in
stationary-phase cells of Saccharomyces cerevisiae.. Proc Natl Acad Sci U S A.

[64] Romano P, Suzzi G (1985). Sensitivity of Saccharomyces cerevisiae vegetative cells and
spores to antimicrobial compounds.. J Appl Bacteriol.

[65] Briza P, Breitenbach M, Ellinger A, Segall J (1990). Isolation of two developmentally regulated genes involved in
spore wall maturation in Saccharomyces cerevisiae.. Genes & Development.

[66] Muthukumar G, Suhng SH, Magee PT, Jewell RD, Primerano DA (1993). The Saccharomyces cerevisiae SPR1 gene encodes a
sporulation-specific exo-1,3-beta-glucanase which contributes to ascospore
thermoresistance.. J Bacteriol.

[67] Reuter M, Bell G, Greig D (2007). Increased outbreeding in yeast in response to dispersal by an
insect vector.. Curr Biol.

[68] Lachance MA, Gilbert DG, Starmer WT (1995). Yeast communities associated with Drosophila species and related
flies in an eastern oak-pine forest: a comparison with western
communities.. J Ind Microbiol.

[69] Rattray A, Santoyo G, Shafer B, Strathern JN (2015). Elevated mutation rate during meiosis in Saccharomyces
cerevisiae.. PLoS Genet.

[70] Primig M, Williams RM, Winzeler EA, Tevzadze GG, Conway AR, Hwang SY, Davis RW, Esposito RE (2000). The core meiotic transcriptome in budding yeasts.. Nat Genet.

[71] Chu S, DeRisi J, Eisen M, Mulholland J, Botstein D, Brown PO, Herskowitz I (1998). The transcriptional program of sporulation in budding
yeast.. Science.

[72] Becker E, Liu Y, Lardenois A, Walther T, Horecka J, Stuparevic I, Law MJ, Lavigne R, Evrard B, Demougin P, Riffle M, Strich R, Davis RW, Pineau C, Primig M (2015). Integrated RNA- and protein profiling of fermentation and
respiration in diploid budding yeast provides insight into nutrient control
of cell growth and development.. Journal of proteomics.

[73] Piccirillo S, Kapros T, Honigberg SM (2016). Phenotypic plasticity within yeast colonies: differential
partitioning of cell fates.. Curr Genet.

[74] Piccirillo S, Morales R, White MG, Smith K, Kapros T, Honigberg SM (2015). Cell Differentiation and Spatial Organization in Yeast Colonies:
Role of Cell-Wall Integrity Pathway.. Genetics.

[75] Esposito RE, Klapholz S (1981). Meiosis and ascospore development.. In: Strathern JN, Jones EW, Broach JR, editors. Molecular biology of the
yeast Saccharomyces: Life cycle and inheritance..

[76] De Silva-Udawatta MN, Cannon JF (2001). Roles of trehalose phosphate synthase in yeast glycogen
metabolism and sporulation.. Molecular microbiology.

[77] Ramaswamy NT, Li L, Khalil M, Cannon JF (1998). Regulation of yeast glycogen metabolism and sporulation by Glc7p
protein phosphatase.. Genetics.

[78] McKnight JN, Boerma JW, Breeden LL, Tsukiyama T (2015). Global Promoter Targeting of a Conserved Lysine Deacetylase for
Transcriptional Shutoff during Quiescence Entry.. Mol Cell.

[79] Miles S, Li L, Davison J, Breeden LL (2013). Xbp1 directs global repression of budding yeast transcription
during the transition to quiescence and is important for the longevity and
reversibility of the quiescent state.. PLoS Genet.

[80] Rutledge MT, Russo M, Belton JM, Dekker J, Broach JR (2015). The yeast genome undergoes significant topological reorganization
in quiescence.. Nucleic Acids Res.

[81] Laporte D, Courtout F, Salin B, Ceschin J, Sagot I (2013). An array of nuclear microtubules reorganizes the budding yeast
nucleus during quiescence.. J Cell Biol.

[82] Munder MC, Midtvedt D, Franzmann T, Nuske E, Otto O, Herbig M, Ulbricht E, Muller P, Taubenberger A, Maharana S, Malinovska L, Richter D, Guck J, Zaburdaev V, Alberti S (2016). A pH-driven transition of the cytoplasm from a fluid- to a
solid-like state promotes entry into dormancy.. Elife.

[83] Joyner RP, Tang JH, Helenius J, Dultz E, Brune C, Holt LJ, Huet S, Muller DJ, Weis K (2016). A glucose-starvation response regulates the diffusion of
macromolecules.. Elife.

[84] Li L, Miles S, Breeden LL (2015). A Genetic Screen for Saccharomyces cerevisiae Mutants That Fail
to Enter Quiescence.. G3 (Bethesda).

[85] Torres J, Di Como CJ, Herrero E, De La Torre-Ruiz MA (2002). Regulation of the cell integrity pathway by rapamycin-sensitive
TOR function in budding yeast.. J Biol Chem.

[86] Cameroni E, Hulo N, Roosen J, Winderickx J, De Virgilio C (2004). The novel yeast PAS kinase Rim 15 orchestrates G0-associated
antioxidant defense mechanisms.. Cell Cycle.

[87] Zhang N, Wu J, Oliver SG (2009). Gis1 is required for transcriptional reprogramming of carbon
metabolism and the stress response during transition into stationary phase
in yeast.. Microbiology.

[88] Roosen J, Engelen K, Marchal K, Mathys J, Griffioen G, Cameroni E, Thevelein JM, De Virgilio C, De Moor B, Winderickx J (2005). PKA and Sch9 control a molecular switch important for the proper
adaptation to nutrient availability.. Mol Microbiol.

[89] Powers 3rd RW, Kaeberlein M, Caldwell SD, Kennedy BK, Fields S (2006). Extension of chronological life span in yeast by decreased TOR
pathway signaling.. Genes and development.

[90] Fabrizio P, Pozza F, Pletcher SD, Gendron CM, Longo VD (2001). Regulation of longevity and stress resistance by Sch9 in
yeast.. Science.

[91] Carmona-Gutierrez D, Buttner S (2014). The many ways to age for a single yeast cell.. Yeast.

[92] Ruckenstuhl C, Carmona-Gutierrez D, Madeo F (2010). The sweet taste of death: glucose triggers apoptosis during yeast
chronological aging.. Aging (Albany NY).

[93] Fabrizio P, Longo VD (2008). Chronological aging-induced apoptosis in yeast.. Biochim Biophys Acta.

[94] Wloch-Salamon DM, Bem AE (2012). Types of cell death and methods of their detection in yeast
Saccharomyces cerevisiae.. J Appl Microbiol.

[95] Strich R (2015). Programmed Cell Death Initiation and Execution in Budding
Yeast.. Genetics.

[96] Greenwood MT, Ludovico P (2010). Expressing and functional analysis of mammalian apoptotic
regulators in yeast.. Cell Death Differ.

[97] Vachova L, Palkova Z (2007). Caspases in yeast apoptosis-like death: facts and
artefacts.. FEMS Yeast Res.

[98] Sinclair D, Mills K, Guarente L (1998). Aging in Saccharomyces cerevisiae.. Annu Rev Microbiol.

[99] Longo VD, Shadel GS, Kaeberlein M, Kennedy B (2012). Replicative and chronological aging in Saccharomyces
cerevisiae.. Cell Metab.

[100] Kaeberlein M, McVey M, Guarente L (2001). Using yeast to discover the fountain of youth.. Sci Aging Knowledge Environ.

[101] Ashrafi K, Sinclair D, Gordon JI, Guarente L (1999). Passage through stationary phase advances replicative aging in
Saccharomyces cerevisiae.. Proc Natl Acad Sci U S A.

[102] Wierman MB, Smith JS (2014). Yeast sirtuins and the regulation of aging.. FEMS Yeast Res.

[103] Gao Q, Ren Q, Liou LC, Bao X, Zhang Z (2011). Mitochondrial DNA protects against salt stress-induced cytochrome
c-mediated apoptosis in yeast.. FEBS letters.

[104] Sapienza K, Bannister W, Balzan R (2008). Mitochondrial involvement in aspirin-induced apoptosis in
yeast.. Microbiology.

[105] Ludovico P, Sousa MJ, Silva MT, Leao C, Corte-Real M (2001). Mitochondrial involvement in aspirin-induced apoptosis in
yeast.. Microbiology.

[106] Madeo F, Frohlich E, Ligr M, Grey M, Sigrist SJ, Wolf DH, Frohlich KU (1999). Oxygen stress: a regulator of apoptosis in yeast.. J Cell Biol.

[107] Longo VD, Fabrizio P (2012). Chronological Aging in Saccharomyces cerevisiae.. Subcell Biochem.

[108] Bishop NA, Guarente L (2007). Genetic links between diet and lifespan: shared mechanisms from
yeast to humans.. Nat Rev Genet.

[109] Lin SJ, Defossez PA, Guarente L (2000). Requirement of NAD and SIR2 for life-span extension by calorie
restriction in Saccharomyces cerevisiae.. Science.

[110] Ayer A, Gourlay CW, Dawes IW (2014). Cellular redox homeostasis, reactive oxygen species and
replicative ageing in Saccharomyces cerevisiae.. FEMS Yeast Res.

[111] Finkel T, Holbrook NJ (2000). Oxidants, oxidative stress and the biology of
ageing.. Nature.

[112] Sohal RS, Weindruch R (1996). Oxidative stress, caloric restriction, and aging.. Science.

[113] Ring J, Sommer C, Carmona-Gutierrez D, Ruckenstuhl C, Eisenberg T, Madeo F (2012). The metabolism beyond programmed cell death in
yeast.. Exp Cell Res.

[114] Ocampo A, Liu J, Schroeder EA, Shadel GS, Barrientos A (2012). Mitochondrial respiratory thresholds regulate yeast chronological
life span and its extension by caloric restriction.. Cell Metab.

[115] Weinberger M, Mesquita A, Caroll T, Marks L, Yang H, Zhang Z, Ludovico P, Burhans WC (2010). Growth signaling promotes chronological aging in budding yeast by
inducing superoxide anions that inhibit quiescence.. Aging (Albany NY).

[116] Fabrizio P, Battistella L, Vardavas R, Gattazzo C, Liou LL, Diaspro A, Dossen JW, Gralla EB, Longo VD (2004). Superoxide is a mediator of an altruistic aging program in
Saccharomyces cerevisiae.. The Journal of cell biology.

[117] Severin FF, Meer MV, Smirnova EA, Knorre DA, Skulachev VP (2008). Natural causes of programmed death of yeast Saccharomyces
cerevisiae.. Biochimica et biophysica acta.

[118] Allocati N, Masulli M, Di Ilio C, De Laurenzi V (2015). Die for the community: an overview of programmed cell death in
bacteria.. Cell Death Dis.

[119] Lewis K (2000). Programmed death in bacteria.. Microbiology and molecular biology reviews : MMBR.

[120] Ameisen JC (1996). The origin of programmed cell death.. Science.

[121] Kron SJ, Styles CA, Fink GR (1994). Symmetric cell division in pseudohyphae of the yeast
Saccharomyces cerevisiae.. Mol Biol Cell.

[122] Dickinson JR (1994). Irreversible formation of pseudohyphae by haploid Saccharomyces
cerevisiae.. FEMS Microbiol Lett.

[123] Kuriyama H, Slaughter JC (1995). Control of cell morphology of the yeast Saccharomyces cerevisiae
by nutrient limitation in continuous culture.. Lett Appl Microbiol.

[124] Pitoniak A, Birkaya B, Dionne HM, Vadaie N, Cullen PJ (2009). The signaling mucins Msb2 and Hkr1 differentially regulate the
filamentation mitogen-activated protein kinase pathway and contribute to a
multimodal response.. Mol Biol Cell.

[125] Martin R, Wachtler B, Schaller M, Wilson D, Hube B (2011). Host-pathogen interactions and virulence-associated genes during
Candida albicans oral infections.. Int J Med Microbiol.

[126] Laxman S, Tu BP (2011). Multiple TORC1-associated proteins regulate nitrogen
starvation-dependent cellular differentiation in Saccharomyces
cerevisiae.. PLoS One.

[127] Ryan O, Shapiro RS, Kurat CF, Mayhew D, Baryshnikova A, Chin B, Lin ZY, Cox MJ, Vizeacoumar F, Cheung D, Bahr S, Tsui K, Tebbji F, Sellam A, Istel F, Schwarzmuller T, Reynolds TB, Kuchler K, Gifford DK, Whiteway M, Giaever G, Nislow C, Costanzo M, Gingras AC, Mitra RD, Andrews B, Fink GR, Cowen LE, Boone C (2012). Global gene deletion analysis exploring yeast filamentous
growth.. Science.

[128] Loeb JD, Kerentseva TA, Pan T, Sepulveda-Becerra M, Liu H (1999). Saccharomyces cerevisiae G1 cyclins are differentially involved
in invasive and pseudohyphal growth independent of the filamentation
mitogen-activated protein kinase pathway.. Genetics.

[129] Lo WS, Dranginis AM (1998). The cell surface flocculin Flo11 is required for pseudohyphae
formation and invasion by Saccharomyces cerevisiae.. Mol Biol Cell.

[130] Voordeckers K, De Maeyer D, van der Zande E, Vinces MD, Meert W, Cloots L, Ryan O, Marchal K, Verstrepen KJ (2012). Identification of a complex genetic network underlying
Saccharomyces cerevisiae colony morphology.. Mol Microbiol.

[131] Granek JA, Magwene PM (2010). Environmental and genetic determinants of colony morphology in
yeast.. PLoS Genet.

[132] Reynolds TB, Fink GR (2001). Bakers' yeast, a model for fungal biofilm
formation.. Science.

[133] Zara G, Zara S, Pinna C, Marceddu S, Budroni M (2009). FLO11 gene length and transcriptional level affect
biofilm-forming ability of wild flor strains of Saccharomyces
cerevisiae.. Microbiology.

[134] Douglas LM, Li L, Yang Y, Dranginis AM (2007). Expression and characterization of the flocculin Flo11/Muc1, a
Saccharomyces cerevisiae mannoprotein with homotypic properties of
adhesion.. Eukaryot Cell.

[135] Cullen PJ, Sprague Jr GF (2012). The regulation of filamentous growth in yeast.. Genetics.

[136] Irniger S (2011). The Ime2 protein kinase family in fungi: more duties than just
meiosis.. Mol Microbiol.

[137] van Werven FJ, Neuert G, Hendrick N, Lardenois A, Buratowski S, van Oudenaarden A, Primig M, Amon A (2012). Transcription of two long noncoding RNAs mediates mating-type
control of gametogenesis in budding yeast.. Cell.

[138] van Dyk D, Hansson G, Pretorius IS, Bauer FF (2003). Cellular differentiation in response to nutrient availability:
The repressor of meiosis, Rme1p, positively regulates invasive growth in
Saccharomyces cerevisiae.. Genetics.

[139] Rupp S, Summers E, Lo HJ, Madhani H, Fink G (1999). MAP kinase and cAMP filamentation signaling pathways converge on
the unusually large promoter of the yeast FLO11 gene.. EMBO J.

[140] Shah JC, Clancy MJ (1992). IME4, a gene that mediates MAT and nutritional control of meiosis
in Saccharomyces cerevisiae.. Mol Cell Biol.

[141] Hongay CF, Grisafi PL, Galitski T, Fink GR (2006). Antisense transcription controls cell fate in Saccharomyces
cerevisiae.. Cell.

[142] Gelfand B, Mead J, Bruning A, Apostolopoulos N, Tadigotla V, Nagaraj V, Sengupta AM, Vershon AK (2011). Regulated antisense transcription controls expression of
cell-type-specific genes in yeast.. Mol Cell Biol.

[143] Agarwala SD, Blitzblau HG, Hochwagen A, Fink GR (2012). RNA methylation by the MIS complex regulates a cell fate decision
in yeast.. PLoS genetics.

[144] Schwartz S, Agarwala SD, Mumbach MR, Jovanovic M, Mertins P, Shishkin A, Tabach Y, Mikkelsen TS, Satija R, Ruvkun G, Carr SA, Lander ES, Fink GR, Regev A (2013). High-resolution mapping reveals a conserved, widespread, dynamic
mRNA methylation program in yeast meiosis.. Cell.

[145] Yue Y, Liu J, He C (2015). RNA N6-methyladenosine methylation in post-transcriptional gene
expression regulation.. Genes Dev.

[146] Uren AG, Beilharz T, O'Connell MJ, Bugg SJ, van Driel R, Vaux DL, Lithgow T (1999). Role for yeast inhibitor of apoptosis (IAP)-like proteins in cell
division.. Proc Natl Acad Sci U S A.

[147] Hurtado S, Kim Guisbert KS, Sontheimer EJ (2014). SPO24 is a transcriptionally dynamic, small ORF-encoding locus
required for efficient sporulation in Saccharomyces
cerevisiae.. PLoS One.

[148] Mai B, Breeden LL (2006). Identification of target genes of a yeast transcriptional
repressor.. Methods Mol Biol.

[149] Mai B, Breeden L (2000). CLN1 and its repression by Xbp1 are important for efficient
sporulation in budding yeast.. Mol Cell Biol.

[150] Purnapatre K, Piccirillo S, Schneider BL, Honigberg SM (2002). The CLN3/SWI6/CLN2 pathway and SNF1 act sequentially to regulate
meiotic initiation in Saccharomyces cerevisiae.. Genes Cells.

[151] Colomina N, Gari E, Gallego C, Herrero E, Aldea M (1999). G1 cyclins block the Ime1 pathway to make mitosis and meiosis
incompatible in budding yeast.. EMBO J.

[152] Aragon AD, Rodriguez AL, Meirelles O, Roy S, Davidson GS, Tapia PH, Allen C, Joe R, Benn D, Werner-Washburne M (2008). Characterization of differentiated quiescent and nonquiescent
cells in yeast stationary-phase cultures.. Mol Biol Cell.

[153] Shi L, Sutter BM, Ye X, Tu BP (2010). Trehalose is a key determinant of the quiescent metabolic state
that fuels cell cycle progression upon return to growth.. Mol Biol Cell.

[154] Tapia H, Morano KA (2010). Hsp90 nuclear accumulation in quiescence is linked to chaperone
function and spore development in yeast.. Mol Biol Cell.

[155] Kurtz S, Rossi J, Petko L, Lindquist S (1986). An ancient developmental induction: heat-shock proteins induced
in sporulation and oogenesis.. Science.

[156] Honigberg SM, Purnapatre K (2003). Signal pathway integration in the switch from the mitotic cell
cycle to meiosis in yeast.. J Cell Sci.

[157] van Werven FJ, Amon A (2011). Regulation of entry into gametogenesis.. Philos Trans R Soc Lond B Biol Sci.

[158] Werner-Washburne M, Roy S, Davidson GS (2012). Aging and the survival of quiescent and non-quiescent cells in
yeast stationary-phase cultures.. Subcell Biochem.

[159] Ceccato-Antonini SR, Sudbery PE (2004). Filamentous growth in Saccharomyces cerevisiae.. Braz J Microbiol.

[160] Jambhekar A, Amon A (2008). Control of meiosis by respiration.. Curr Biol.

[161] Lee RH, Honigberg SM (1996). Nutritional regulation of late meiotic events in Saccharomyces
cerevisiae through a pathway distinct from initiation.. Mol Cell Biol.

[162] Conway MK, Grunwald D, Heideman W (2012). Glucose, nitrogen, and phosphate repletion in Saccharomyces
cerevisiae: common transcriptional responses to different nutrient
signals.. G3 (Bethesda).

[163] Galdieri L, Mehrotra S, Yu S, Vancura A (2010). Transcriptional regulation in yeast during diauxic shift and
stationary phase.. Omics.

[164] Klosinska MM, Crutchfield CA, Bradley PH, Rabinowitz JD, Broach JR (2011). Yeast cells can access distinct quiescent states.. Genes Dev.

[165] Orlandi I, Ronzulli R, Casatta N, Vai M (2013). Ethanol and acetate acting as carbon/energy sources negatively
affect yeast chronological aging.. Oxid Med Cell Longev.

[166] Burtner CR, Murakami CJ, Kennedy BK, Kaeberlein M (2009). A molecular mechanism of chronological aging in
yeast.. Cell Cycle.

[167] Palecek SP, Parikh AS, Huh JH, Kron SJ (2002). Depression of Saccharomyces cerevisiae invasive growth on
non-glucose carbon sources requires the Snf1 kinase.. Molecular microbiology.

[168] Cullen PJ, Sprague Jr GF (2002). The roles of bud-site-selection proteins during haploid invasive
growth in yeast.. Molecular biology of the cell.

[169] Cullen PJ (2015). Evaluating yeast filamentous growth at the single-cell
level.. Cold Spring Harbor protocols.

[170] Godard P, Urrestarazu A, Vissers S, Kontos K, Bontempi G, van Helden J, Andre B (2007). Effect of 21 different nitrogen sources on global gene expression
in the yeast Saccharomyces cerevisiae.. Mol Cell Biol.

[171] Day A, Markwardt J, Delaguila R, Zhang J, Purnapatre K, Honigberg SM, Schneider BL (2004). Cell size and Cln-Cdc28 complexes mediate entry into meiosis by
modulating cell growth.. Cell Cycle.

[172] Colomina N, Liu Y, Aldea M, Gari E (2003). TOR regulates the subcellular localization of Ime1, a
transcriptional activator of meiotic development in budding
yeast.. Mol Cell Biol.

[173] Santos J, Leitao-Correia F, Sousa MJ, Leao C (2015). Ammonium is a key determinant on the dietary restriction of yeast
chronological aging in culture medium.. Oncotarget.

[174] Vinod PK, Sengupta N, Bhat PJ, Venkatesh KV (2008). Integration of global signaling pathways, cAMP-PKA, MAPK and TOR
in the regulation of FLO11.. PLoS One.

[175] Boeckstaens M, Andre B, Marini AM (2007). The yeast ammonium transport protein Mep2 and its positive
regulator, the Npr1 kinase, play an important role in normal and
pseudohyphal growth on various nitrogen media through retrieval of excreted
ammonium.. Mol Microbiol.

[176] Murray LE, Rowley N, Dawes IW, Johnston GC, Singer RA (1998). A yeast glutamine tRNA signals nitrogen status for regulation of
dimorphic growth and sporulation.. Proc Natl Acad Sci U S A.

[177] Lorenz MC, Heitman J (1998). The MEP2 ammonium permease regulates pseudohyphal differentiation
in Saccharomyces cerevisiae.. EMBO J.

[177] Orij R, Postmus J, Ter Beek A, Brul S, Smits GJ (2009). In vivo measurement of cytosolic and mitochondrial pH using a
pH-sensitive GFP derivative in Saccharomyces cerevisiae reveals a relation
between intracellular pH and growth.. EMBO J.

[179] Murakami C, Delaney JR, Chou A, Carr D, Schleit J, Sutphin GL, An EH, Castanza AS, Fletcher M, Goswami S, Higgins S, Holmberg M, Hui J, Jelic M, Jeong KS, Kim JR, Klum S, Liao E, Lin MS, Lo W, Miller H, Moller R, Peng ZJ, Pollard T, Pradeep P, Pruett D, Rai D, Ros V, Schuster A, Singh M (2012). pH neutralization protects against reduction in replicative
lifespan following chronological aging in yeast.. Cell Cycle.

[180] Hayashi M, Ohkuni K, Yamashita I (1998). Control of division arrest and entry into meiosis by
extracellular alkalization in Saccharomyces cerevisiae.. Yeast.

[181] Mirisola MG, Longo VD (2012). Acetic acid and acidification accelerate chronological and
replicative aging in yeast.. Cell Cycle.

[182] Piccirillo S, White MG, Murphy JC, Law DJ, Honigberg SM (2010). The Rim101p/PacC pathway and alkaline pH regulate pattern
formation in yeast colonies.. Genetics.

[183] Ohkuni K, Hayashi M, Yamashita I (1998). Bicarbonate-mediated social communication stimulates meiosis and
sporulation of Saccharomyces cerevisiae.. Yeast.

[184] Soares EV (2011). Flocculation in Saccharomyces cerevisiae: a
review.. J Appl Microbiol.

[185] Stratford M (1996). Induction of flocculation in brewing yeasts by change in pH
value.. FEMS Microbiol Lett.

[186] Li W, Mitchell AP (1997). Proteolytic activation of Rim1p, a positive regulator of yeast
sporulation and invasive growth.. Genetics.

[187] Esposito MS, Esposito RE (1969). The genetic control of sporulation in Saccharomyces. I. The
isolation of temperature-sensitive sporulation-deficient
mutants.. Genetics.

[188] McCusker JH, Clemons KV, Stevens DA, Davis RW (1994). Saccharomyces cerevisiae virulence phenotype as determined with
CD-1 mice is associated with the ability to grow at 42 degrees C and form
pseudohyphae.. Infect Immun.

[189] McCusker JH, Clemons KV, Stevens DA, Davis RW (1994). Genetic characterization of pathogenic Saccharomyces cerevisiae
isolates.. Genetics.

[190] Nussbaum I, Weindling E, Jubran R, Cohen A, Bar-Nun S (2014). Deteriorated stress response in stationary-phase yeast: Sir2 and
Yap1 are essential for Hsf1 activation by heat shock and oxidative stress,
respectively.. PLoS One.

[191] Kourtis N, Tavernarakis N (2011). Cellular stress response pathways and ageing: intricate molecular
relationships.. EMBO J.

[192] Verstrepen KJ, Fink GR (2009). Genetic and epigenetic mechanisms underlying cell-surface
variability in protozoa and fungi.. Annu Rev Genet.

[193] Cullen PJ (2007). Signaling mucins: the new kids on the MAPK block.. Crit Rev Eukaryot Gene Expr.

[194] Conrad M, Schothorst J, Kankipati HN, Van Zeebroeck G, Rubio-Texeira M, Thevelein JM (2014). Nutrient sensing and signaling in the yeast Saccharomyces
cerevisiae.. FEMS Microbiol Rev.

[195] Broach JR (2012). Nutritional control of growth and development in
yeast.. Genetics.

[196] Hedbacker K, Carlson M (2008). SNF1/AMPK pathways in yeast.. Front Biosci.

[197] Orlova M, Ozcetin H, Barrett L, Kuchin S (2010). Roles of the Snf1-activating kinases during nitrogen limitation
and pseudohyphal differentiation in Saccharomyces
cerevisiae.. Eukaryot Cell.

[198] Hughes Hallett JE, Luo X, Capaldi AP (2014). State transitions in the TORC1 signaling pathway and information
processing in Saccharomyces cerevisiae.. Genetics.

[199] Thevelein JM, Bonini BM, Castermans D, Haesendonckx S, Kriel J, Louwet W, Thayumanavan P, Popova Y, Rubio-Texeira M, Schepers W, Vandormael P, Van Zeebroeck G, Verhaert P, Versele M, Voordeckers K (2008). Novel mechanisms in nutrient activation of the yeast protein
kinase A pathway.. Acta Microbiol Immunol Hung.

[200] Jungbluth M, Mosch HU, Taxis C (2012). Acetate Regulation of Spore Formation Is under the Control of the
Ras/Cyclic AMP/Protein Kinase A Pathway and Carbon Dioxide in Saccharomyces
cerevisiae.. Eukaryotic cell.

[201] Dechant R, Saad S, Ibanez AJ, Peter M (2014). Cytosolic pH regulates cell growth through distinct GTPases, Arf1
and Gtr1, to promote Ras/PKA and TORC1 activity.. Mol Cell.

[202] Dechant R, Binda M, Lee SS, Pelet S, Winderickx J, Peter M (2010). Cytosolic pH is a second messenger for glucose and regulates the
PKA pathway through V-ATPase.. EMBO J.

[203] Mizunuma M, Tsubakiyama R, Ogawa T, Shitamukai A, Kobayashi Y, Inai T, Kume K, Hirata D (2013). Ras/cAMP-dependent protein kinase (PKA) regulates multiple
aspects of cellular events by phosphorylating the Whi3 cell cycle regulator
in budding yeast.. J Biol Chem.

[204] Burtner CR, Murakami CJ, Olsen B, Kennedy BK, Kaeberlein M (2011). A genomic analysis of chronological longevity factors in budding
yeast.. Cell Cycle.

[205] Mosch HU, Fink GR (1997). Dissection of filamentous growth by transposon mutagenesis in
Saccharomyces cerevisiae.. Genetics.

[206] Reinders A, Burckert N, Boller T, Wiemken A, De Virgilio C (1998). Saccharomyces cerevisiae cAMP-dependent protein kinase controls
entry into stationary phase through the Rim15p protein
kinase.. Genes and development.

[207] Watanabe D, Zhou Y, Hirata A, Sugimoto Y, Takagi K, Akao T, Ohya Y, Takagi H, Shimoi H (2015). Inhibitory Role of Greatwall-Like Protein Kinase Rim15p in
Alcoholic Fermentation via Upregulating the UDP-Glucose Synthesis Pathway in
Saccharomyces cerevisiae.. Appl Environ Microbiol.

[208] Bontron S, Jaquenoud M, Vaga S, Talarek N, Bodenmiller B, Aebersold R, De Virgilio C (2013). Yeast endosulfines control entry into quiescence and
chronological life span by inhibiting protein phosphatase
2A.. Cell Rep.

[209] Luo X, Talarek N, Virgilio CD (2011). Initiation of the yeast G 0 program requires Igo1 and Igo2, which
antagonize activation of decapping of specific nutrient-regulated
mRNAs.. RNA Biol.

[210] Talarek N, Cameroni E, Jaquenoud M, Luo X, Bontron S, Lippman S, Devgan G, Snyder M, Broach JR, De Virgilio C (2010). Initiation of the TORC1-regulated G0 program requires Igo1/2,
which license specific mRNAs to evade degradation via the 5'-3' mRNA decay
pathway.. Mol Cell.

[211] Lee P, Kim MS, Paik SM, Choi SH, Cho BR, Hahn JS (2013). Rim15-dependent activation of Hsf1 and Msn2/4 transcription
factors by direct phosphorylation in Saccharomyces
cerevisiae.. FEBS Lett.

[212] Wei M, Fabrizio P, Hu J, Ge H, Cheng C, Li L, Longo VD (2008). Life span extension by calorie restriction depends on Rim15 and
transcription factors downstream of Ras/PKA, Tor, and Sch9.. PLoS genetics.

[213] Vidan S, Mitchell AP (1997). Stimulation of yeast meiotic gene expression by the
glucose-repressible protein kinase Rim15p.. Mol Cell Biol.

[214] Quan Z, Cao L, Tang Y, Yan Y, Oliver SG, Zhang N (2015). The Yeast GSK-3 Homologue Mck1 Is a Key Controller of Quiescence
Entry and Chronological Lifespan.. PLoS Genet.

[215] Swinnen E, Wanke V, Roosen J, Smets B, Dubouloz F, Pedruzzi I, Cameroni E, De Virgilio C, Winderickx J (2006). Rim15 and the crossroads of nutrient signalling pathways in
Saccharomyces cerevisiae.. Cell Div.

[216] Sarkar S, Dalgaard JZ, Millar JB, Arumugam P (2014). The Rim15-endosulfine-PP2ACdc55 signalling module regulates entry
into gametogenesis and quiescence via distinct mechanisms in budding
yeast.. PLoS Genet.

[217] Ward MP, Gimeno CJ, Fink GR, Garrett S (1995). SOK2 may regulate cyclic AMP-dependent protein kinase-stimulated
growth and pseudohyphal development by repressing
transcription.. Mol Cell Biol.

[218] Ward MP, Garrett S (1994). Suppression of a yeast cyclic AMP-dependent protein kinase defect
by overexpression of SOK1, a yeast gene exhibiting sequence similarity to a
developmentally regulated mouse gene.. Mol Cell Biol.

[219] Shenhar G, Kassir Y (2001). A positive regulator of mitosis, Sok2, functions as a negative
regulator of meiosis in Saccharomyces cerevisiae.. Mol Cell Biol.

[220] Pan X, Heitman J (2000). Sok2 regulates yeast pseudohyphal differentiation via a
transcription factor cascade that regulates cell-cell
adhesion.. Mol Cell Biol.

[221] Borneman AR, Leigh-Bell JA, Yu H, Bertone P, Gerstein M, Snyder M (2006). Target hub proteins serve as master regulators of development in
yeast.. Genes Dev.

[222] McDonald CM, Wagner M, Dunham MJ, Shin ME, Ahmed NT, Winter E (2009). The Ras/cAMP pathway and the CDK-like kinase Ime2 regulate the
MAPK Smk1 and spore morphogenesis in Saccharomyces
cerevisiae.. Genetics.

[223] Lee P, Paik SM, Shin CS, Huh WK, Hahn JS (2011). Regulation of yeast Yak1 kinase by PKA and
autophosphorylation-dependent 14-3-3 binding.. Mol Microbiol.

[224] Lee P, Cho BR, Joo HS, Hahn JS (2008). Yeast Yak1 kinase, a bridge between PKA and stress-responsive
transcription factors, Hsf1 and Msn2/Msn4.. Mol Microbiol.

[225] Cardona F, Del Olmo ML, Aranda A (2012). Phylogenetic origin and transcriptional regulation at the
post-diauxic phase of SPI1, in Saccharomyces cerevisiae.. Cell Mol Biol Lett.

[226] Werner-Washburne M, Brown D, Braun E (1991). Bcy1, the regulatory subunit of cAMP-dependent protein kinase in
yeast, is differentially modified in response to the physiological status of
the cell.. J Biol Chem.

[227] Malcher M, Schladebeck S, Mosch HU (2011). The Yak1 protein kinase lies at the center of a regulatory
cascade affecting adhesive growth and stress resistance in Saccharomyces
cerevisiae.. Genetics.

[228] Mirisola MG, Taormina G, Fabrizio P, Wei M, Hu J, Longo VD (2014). Serine- and threonine/valine-dependent activation of PDK and Tor
orthologs converge on Sch9 to promote aging.. PLoS Genet.

[229] Swinnen E, Ghillebert R, Wilms T, Winderickx J (2014). Molecular mechanisms linking the evolutionary conserved
TORC1-Sch9 nutrient signalling branch to lifespan regulation in
Saccharomyces cerevisiae.. FEMS Yeast Res.

[230] Weidberg H, Moretto F, Spedale G, Amon A, van Werven FJ (2016). Nutrient Control of Yeast Gametogenesis Is Mediated by TORC1, PKA
and Energy Availability.. PLoS Genet.

[231] Pan Y, Schroeder EA, Ocampo A, Barrientos A, Shadel GS (2011). Regulation of yeast chronological life span by TORC1 via adaptive
mitochondrial ROS signaling.. Cell Metab.

[232] Pan Y, Shadel GS (2009). Extension of chronological life span by reduced TOR signaling
requires down-regulation of Sch9p and involves increased mitochondrial
OXPHOS complex density.. Aging (Albany NY).

[233] Cutler NS, Pan X, Heitman J, Cardenas ME (2001). The TOR signal transduction cascade controls cellular
differentiation in response to nutrients.. Molecular biology of the cell.

[234] Bruckner S, Kern S, Birke R, Saugar I, Ulrich HD, Mosch HU (2011). The TEA transcription factor Tec1 links TOR and MAPK pathways to
coordinate yeast development.. Genetics.

[235] Humston EM, Dombek KM, Tu BP, Young ET, Synovec RE (2011). Toward a global analysis of metabolites in regulatory mutants of
yeast.. Anal Bioanal Chem.

[236] Usaite R, Jewett MC, Oliveira AP, Yates 3rd JR, Olsson L, Nielsen J (2009). Reconstruction of the yeast Snf1 kinase regulatory network
reveals its role as a global energy regulator.. Mol Syst Biol.

[237] Wang Z, Wilson WA, Fujino MA, Roach PJ (2001). Antagonistic controls of autophagy and glycogen accumulation by
Snf1p, the yeast homolog of AMP-activated protein kinase, and the
cyclin-dependent kinase Pho85p.. Mol Cell Biol.

[238] Enjalbert B, Parrou JL, Teste MA, Francois J (2004). Combinatorial control by the protein kinases PKA, PHO85 and SNF1
of transcriptional induction of the Saccharomyces cerevisiae GSY2 gene at
the diauxic shift.. Mol Genet Genomics.

[239] Van de Velde S, Thevelein JM (2008). Cyclic AMP-protein kinase A and Snf1 signaling mechanisms
underlie the superior potency of sucrose for induction of filamentation in
Saccharomyces cerevisiae.. Eukaryot Cell.

[240] Kuchin S, Vyas VK, Carlson M (2002). Snf1 protein kinase and the repressors Nrg1 and Nrg2 regulate
FLO11, haploid invasive growth, and diploid pseudohyphal
differentiation.. Mol Cell Biol.

[241] Lorenz DR, Cantor CR, Collins JJ (2009). A network biology approach to aging in yeast.. Proc Natl Acad Sci U S A.

[242] Ashrafi K, Lin SS, Manchester JK, Gordon JI (2000). Sip2p and its partner snf1p kinase affect aging in S.
cerevisiae.. Genes Dev.

[243] Maeda T (2012). The signaling mechanism of ambient pH sensing and adaptation in
yeast and fungi.. Febs J.

[244] Scherz Andersen K, Bojsen R, Gro Rejkjaer Sorensen L, Weiss Nielsen M, Lisby M, Folkesson A, Regenberg B (2014). Genetic basis for Saccharomyces cerevisiae biofilm in liquid
medium.. G3 (Bethesda).

[245] Chavel CA, Dionne HM, Birkaya B, Joshi J, Cullen PJ (2010). Multiple signals converge on a differentiation MAPK
pathway.. PLoS Genet.

[246] Lamb TM, Mitchell AP (2003). The transcription factor Rim101p governs ion tolerance and cell
differentiation by direct repression of the regulatory genes NRG1 and SMP1
in Saccharomyces cerevisiae.. Mol Cell Biol.

[247] Read T, Richmond PA, Dowell RD (2016). A trans-acting Variant within the Transcription Factor RIM101
Interacts with Genetic Background to Determine its Regulatory
Capacity.. PLoS Genet.

[248] Gray M, Piccirillo S, Purnapatre K, Schneider BL, Honigberg SM (2008). Glucose induction pathway regulates meiosis in Saccharomyces
cerevisiae in part by controlling turnover of Ime2p meiotic
kinase.. FEMS Yeast Res.

[249] Purnapatre K, Gray M, Piccirillo S, Honigberg SM (2005). Glucose inhibits meiotic DNA replication through
SCFGrr1p-dependent destruction of Ime2p kinase.. Molecular and cellular biology.

[250] Shively CA, Eckwahl MJ, Dobry CJ, Mellacheruvu D, Nesvizhskii A, Kumar A (2013). Genetic networks inducing invasive growth in Saccharomyces
cerevisiae identified through systematic genome-wide
overexpression.. Genetics.

[251] Furukawa K, Furukawa T, Hohmann S (2011). Efficient construction of homozygous diploid strains identifies
genes required for the hyper-filamentous phenotype in Saccharomyces
cerevisiae.. PLoS One.

[252] Krause SA, Gray JV (2002). The protein kinase C pathway is required for viability in
quiescence in Saccharomyces cerevisiae.. Current biology : CB.

[253] Sundaram V, Petkova MI, Pujol-Carrion N, Boada J, de la Torre-Ruiz MA (2015). Tor1, Sch9 and PKA downregulation in quiescence rely on Mtl1 to
preserve mitochondrial integrity and cell survival.. Mol Microbiol.

[254] Vancetto GT, Ceccato-Antonini SR (2007). MPK1 gene is required for filamentous growth induced by isoamyl
alcohol in Saccharomyces cerevisiae strains from the alcoholic
fermentation.. Appl Microbiol Biotechnol.

[255] Martinez de Maranon I, Marechal PA, Gervais P (1996). Passive response of Saccharomyces cerevisiae to osmotic shifts:
cell volume variations depending on the physiological state.. Biochem Biophys Res Commun.

[256] de Llanos R, Hernandez-Haro C, Barrio E, Querol A, Fernandez-Espinar MT, Molina M (2010). Differences in activation of MAP kinases and variability in the
polyglutamine tract of Slt2 in clinical and non-clinical isolates of
Saccharomyces cerevisiae.. Yeast.

[257] Schroder M, Clark R, Liu CY, Kaufman RJ (2004). The unfolded protein response represses differentiation through
the RPD3-SIN3 histone deacetylase.. EMBO J.

[258] Orlova M, Kanter E, Krakovich D, Kuchin S (2006). Nitrogen availability and TOR regulate the Snf1 protein kinase in
Saccharomyces cerevisiae.. Eukaryot Cell.

[259] Nishizawa M, Tanigawa M, Hayashi M, Maeda T, Yazaki Y, Saeki Y, Toh-e A (2010). Pho85 kinase, a cyclin-dependent kinase, regulates nuclear
accumulation of the Rim101 transcription factor in the stress response of
Saccharomyces cerevisiae.. Eukaryot Cell.

[260] Law MJ, Ciccaglione K (2015). Fine-tuning of histone H3 Lys4 methylation during pseudohyphal
differentiation by the CDK submodule of RNA polymerase II.. Genetics.

[261] Halme A, Bumgarner S, Styles C, Fink GR (2004). Genetic and epigenetic regulation of the FLO gene family
generates cell-surface variation in yeast.. Cell.

[262] Wang LC, Montalvo-Munoz F, Tsai YC, Liang CY, Chang CC, Lo WS (2015). The Histone Acetyltransferase Gcn5 Regulates ncRNA-ICR1 and FLO11
Expression during Pseudohyphal Development in Saccharomyces
cerevisiae.. Biomed Res Int.

[263] Bumgarner SL, Neuert G, Voight BF, Symbor-Nagrabska A, Grisafi P, van Oudenaarden A, Fink GR (2012). Single-cell analysis reveals that noncoding RNAs contribute to
clonal heterogeneity by modulating transcription factor
recruitment.. Mol Cell.

[264] Bumgarner SL, Dowell RD, Grisafi P, Gifford DK, Fink GR (2009). Toggle involving cis-interfering noncoding RNAs controls
variegated gene expression in yeast.. Proc Natl Acad Sci U S A.

[265] Holmes DL, Lancaster AK, Lindquist S, Halfmann R (2013). Heritable remodeling of yeast multicellularity by an
environmentally responsive prion.. Cell.

[266] Lardenois A, Stuparevic I, Liu Y, Law MJ, Becker E, Smagulova F, Waern K, Guilleux MH, Horecka J, Chu A, Kervarrec C, Strich R, Snyder M, Davis RW, Steinmetz LM, Primig M (2015). The conserved histone deacetylase Rpd3 and its DNA binding
subunit Ume6 control dynamic transcript architecture during mitotic growth
and meiotic development.. Nucleic Acids Res.

[267] Krishnamoorthy T, Chen X, Govin J, Cheung WL, Dorsey J, Schindler K, Winter E, Allis CD, Guacci V, Khochbin S, Fuller MT, Berger SL (2006). Phosphorylation of histone H4 Ser1 regulates sporulation in yeast
and is conserved in fly and mouse spermatogenesis.. Genes Dev.

[268] Xu M, Soloveychik M, Ranger M, Schertzberg M, Shah Z, Raisner R, Venkatasubrahmanyan S, Tsui K, Gebbia M, Hughes T, van Bakel H, Nislow C, Madhani HD, Meneghini MD (2012). Timing of transcriptional quiescence during gametogenesis is
controlled by global histone H3K4 demethylation.. Dev Cell.

[269] McKnight JN, Tsukiyama T (2015). The conserved HDAC Rpd3 drives transcriptional quiescence in S.
cerevisiae.. Genom Data.

[270] Radonjic M, Andrau JC, Lijnzaad P, Kemmeren P, Kockelkorn TT, van Leenen D, van Berkum NL, Holstege FC (2005). Genome-wide analyses reveal RNA polymerase II located upstream of
genes poised for rapid response upon S. cerevisiae stationary phase
exit.. Mol Cell.

[271] Ganesan AT, Holter H, Roberts C (1958). Some observations on sporulation in
Saccharomyces.. CR Lab Carlsberg.

[272] Kirsop BH (1954). Studies in yeast sporulation. I. Some factors influencing
sporulation.. J Inst Brew.

[273] Tsuchiya D, Yang Y, Lacefield S (2014). Positive feedback of NDT80 expression ensures irreversible
meiotic commitment in budding yeast.. PLoS Genet.

[274] Winter E (2012). The Sum1/Ndt80 transcriptional switch and commitment to meiosis
in Saccharomyces cerevisiae.. Microbiology and molecular biology reviews : MMBR.

[275] Corbi D, Sunder S, Weinreich M, Skokotas A, Johnson ES, Winter E (2014). Multisite phosphorylation of the Sum1 transcriptional repressor
by S-phase kinases controls exit from meiotic prophase in
yeast.. Mol Cell Biol.

[276] Berchowitz LE, Kabachinski G, Walker MR, Carlile TM, Gilbert WV, Schwartz TU, Amon A (2015). Regulated Formation of an Amyloid-like Translational Repressor
Governs Gametogenesis.. Cell.

[277] Jin L, Zhang K, Xu Y, Sternglanz R, Neiman AM (2015). Sequestration of mRNAs Modulates the Timing of Translation during
Meiosis in Budding Yeast.. Mol Cell Biol.

[278] Honigberg SM, Conicella C, Espositio RE (1992). Commitment to meiosis in Saccharomyces cerevisiae: involvement of
the SPO14 gene.. Genetics.

[279] Honigberg SM, Esposito RE (1994). Reversal of cell determination in yeast meiosis: postcommitment
arrest allows return to mitotic growth.. Proceedings of the National Academy of Sciences of the United States of
America.

[280] Yang H, Ren Q, Zhang Z (2006). Chromosome or chromatin condensation leads to meiosis or
apoptosis in stationary yeast (Saccharomyces cerevisiae)
cells.. FEMS yeast research.

[281] Davidson GS, Joe RM, Roy S, Meirelles O, Allen CP, Wilson MR, Tapia PH, Manzanilla EE, Dodson AE, Chakraborty S, Carter M, Young S, Edwards B, Sklar L, Werner-Washburne M (2011). The proteomics of quiescent and nonquiescent cell differentiation
in yeast stationary-phase cultures.. Mol Biol Cell.

[282] Allen C, Buttner S, Aragon AD, Thomas JA, Meirelles O, Jaetao JE, Benn D, Ruby SW, Veenhuis M, Madeo F, Werner-Washburne M (2006). Isolation of quiescent and nonquiescent cells from yeast
stationary-phase cultures.. J Cell Biol.

[283] Palkova Z, Vachova L (2016). Yeast cell differentiation: Lessons from pathogenic and
non-pathogenic yeasts.. Semin Cell Dev Biol.

[284] Honigberg SM (2011). Cell signals, cell contacts, and the organization of yeast
communities.. Eukaryot Cell.

[285] Piccirillo S, Honigberg SM (2011). Yeast colony embedding method.. J Vis Exp.

[286] Vachova L, Hatakova L, Cap M, Pokorna M, Palkova Z (2013). Rapidly developing yeast microcolonies differentiate in a similar
way to aging giant colonies.. Oxid Med Cell Longev.

[287] Cap M, Stepanek L, Harant K, Vachova L, Palkova Z (2012). Cell differentiation within a yeast colony: metabolic and
regulatory parallels with a tumor-affected organism.. Mol Cell.

[288] Baillie GS, Douglas LJ (1999). Role of dimorphism in the development of Candida albicans
biofilms.. J Med Microbiol.

[289] Chandra J, Kuhn DM, Mukherjee PK, Hoyer LL, McCormick T, Ghannoum MA (2001). Biofilm formation by the fungal pathogen Candida albicans:
development, architecture, and drug resistance.. J Bacteriol.

[290] Vachova L, Palkova Z (2005). Physiological regulation of yeast cell death in multicellular
colonies is triggered by ammonia.. J Cell Biol.

[291] Vachova L, Palkova Z (2011). Aging and longevity of yeast colony populations: metabolic
adaptation and differentiation.. Biochem Soc Trans.

[292] Chen H, Fink GR (2006). Feedback control of morphogenesis in fungi by aromatic
alcohols.. Genes Dev.

[293] Vachova L, Kucerova H, Devaux F, Ulehlova M, Palkova Z (2009). Metabolic diversification of cells during the development of
yeast colonies.. Environ Microbiol.

[294] Vachova L, Chernyavskiy O, Strachotova D, Bianchini P, Burdikova Z, Fercikova I, Kubinova L, Palkova Z (2009). Architecture of developing multicellular yeast colony:
spatio-temporal expression of Ato1p ammonium exporter.. Environ Microbiol.

[295] Palkova Z, Devaux F, Icicova M, Minarikova L, Le Crom S, Jacq C (2002). Ammonia pulses and metabolic oscillations guide yeast colony
development.. Mol Biol Cell.

[296] Palkova Z, Forstova J (2000). Yeast colonies synchronise their growth and
development.. J Cell Sci.

[297] Gordon DM (2013). The rewards of restraint in the collective regulation of foraging
by harvester ant colonies.. Nature.

[298] Wilson EO, Holldobler B (1988). Dense heterarchies and mass communication as the basis of
organization in ant colonies.. Trends Ecol Evol.

[299] Cap M, Vachova L, Palkova Z (2012). Reactive oxygen species in the signaling and adaptation of
multicellular microbial communities.. Oxid Med Cell Longev.

[300] Cap M, Vachova L, Palkova Z (2009). Yeast colony survival depends on metabolic adaptation and cell
differentiation rather than on stress defense.. J Biol Chem.

[301] St Johnston D, Sanson B (2011). Epithelial polarity and morphogenesis.. Curr Opin Cell Biol.

[302] Irvine KD, Rauskolb C (2001). Boundaries in development: formation and
function.. Annu Rev Cell Dev Biol.

[303] Rokas A (2008). The origins of multicellularity and the early history of the
genetic toolkit for animal development.. Annu Rev Genet.

[304] Hall-Stoodley L, Costerton JW, Stoodley P (2004). Bacterial biofilms: from the natural environment to infectious
diseases.. Nat Rev Microbiol.

[305] Niklas KJ, Newman SA (2013). The origins of multicellular organisms.. Evol Dev.

[306] Knoll AH (2011). The multiple origins of complex multicellularity.. Annu Rev Earth Planet Sci.

